# Environmental Drivers of the Canadian Arctic Megabenthic Communities

**DOI:** 10.1371/journal.pone.0100900

**Published:** 2014-07-14

**Authors:** Virginie Roy, Katrin Iken, Philippe Archambault

**Affiliations:** 1 Institut des sciences de la mer de Rimouski, Université du Québec à Rimouski, Rimouski, Québec, Canada; 2 School of Fisheries and Ocean Sciences, University of Alaska Fairbanks, Fairbanks, Alaska, United States of America; University of Waikato (National Institute of Water and Atmospheric Research), New Zealand

## Abstract

Environmental gradients and their influence on benthic community structure vary over different spatial scales; yet, few studies in the Arctic have attempted to study the influence of environmental gradients of differing spatial scales on megabenthic communities across continental-scales. The current project studied for the first time how megabenthic community structure is related to several environmental factors over 2000 km of the Canadian Arctic, from the Beaufort Sea to northern Baffin Bay. Faunal trawl samples were collected between 2007 and 2011 at 78 stations from 30 to 1000 m depth and patterns in biomass, density, richness, diversity, and taxonomic composition were examined in relation to indirect/spatial gradients (e.g., depth), direct gradients (e.g., bottom oceanographic variables), and resource gradients (e.g., food supply proxies). Six benthic community types were defined based on their biomass-based taxonomic composition. Their distribution was significantly, but moderately, associated with large-scale (100–1000 km) environmental gradients defined by depth, physical water properties (e.g., bottom salinity), and meso-scale (10–100 km) environmental gradients defined by substrate type (hard vs. soft) and sediment organic carbon content. We did not observe a strong decline of bulk biomass, density and richness with depth or a strong increase of those community characteristics with food supply proxies, contrary to our hypothesis. We discuss how local- to meso-scale environmental conditions, such as bottom current regimes and polynyas, sustain biomass-rich communities at specific locations in oligotrophic and in deep regions of the Canadian Arctic. This study demonstrates the value of considering the scales of variability of environmental gradients when interpreting their relevance in structuring of communities.

## Introduction

In Arctic systems, megabenthic communities contribute significantly to bulk benthic biomass [1], [2] with high oxygen demands [1], [3]–[5] and important roles in carbon cycling on Arctic shelves [6], [7]. Megabenthic communities also provide an important link to higher trophic levels as food for many sea birds and marine mammals [8], [9]. Despite their importance in Arctic food webs, little is still known, however, about their distributional patterns and the environmental factors driving them across the large spatial extents, such as the Canadian Arctic Archipelago.

The external drivers of benthic community dynamics change with the spatial scale under investigation. At small scales (e.g., within a sampling station), community structure is controlled mainly by ecological factors such as the availability of niches superimposed by competition and predation, while at meso (10–100 km) to large (100–1000 km) scales it is mainly controlled by environmental gradients [10]–[13]. Depth and geographic gradients generate large-scale benthic patterns that are well known in the World's oceans [14], [15]. For Arctic megafaunal communities, depth is often considered one of the most important large-scale structuring variables [1], [16]–[20]. However, depth is mostly a proxy of other environmental variables that vary vertically, such as physical properties of water masses (temperature, salinity) and declining food availability for slope and deep-sea benthic communities [21]. In the highly seasonal Arctic systems, the declining strength of pelagic-benthic coupling and the resultant reduced food supply is thought to be the most important indirect effect of depth in structuring benthic communities [7], [18] and benthic processes [22]. In contrast to large-scale gradients, patterns in current regimes and sea-ice cover, by their influence on primary production and on the sedimentation of organic matter out of the water column, produce meso-scale benthic patterns that are typically regionally specific, such as under polynyas and marginal ice zones in the Arctic [13]. In the quest to elucidate the importance of food supply on Arctic benthic communities, and because of the complexity of biological and physical interactions that can increase or reduce pelagic-benthic coupling, various food supply proxies are often used to interpret benthic community patterns [23]. This study tested a variety of food supply proxies, from estimates of particulate organic carbon (POC) fluxes (e.g., derived from primary productivity in surface waters) to estimates of available organic matter for benthic organisms (e.g., sediment pigment). Substrate variability is also an important local- to meso-scale driver of megabenthic taxonomic composition in both Arctic shelf and slope regions [2], [24]. By reflecting near-bottom flow regime, substrate variability influences benthic feeding modes and survival of organisms due to specific requirements from larvae to adult stages [25], and thus profoundly affects benthic community composition.

The Canadian Arctic is an excellent candidate area to test whether large-scale and meso-scale environment-benthic community relationships found elsewhere across the World's oceans also apply within a large, topographically and hydrographically complex Arctic marine environment. The Canadian Arctic is characterized by great depth variation, complex flow dynamics [26], contrasting biological productivity regimes [27], and significant freshwater and sediment inflow from the Mackenzie River, by far the most sediment-rich river discharging into the Arctic Ocean [28].

The current project studied how megabenthic community structure is associated with environmental gradients across 70° longitude (2000 km) of the Canadian Arctic marine environment. The specific objectives of this study were: (1) to delineate community clusters and characterize their structure and distribution patterns, and (2) to evaluate the relationships of environmental factors of various spatial scales with megabenthic community characteristics (e.g., richness, biomass) and community distribution. We hypothesized that: (i) megabenthic biomass, density, richness and diversity decrease with depth and increase with food supply proxies, and (ii) community patterns are associated primarily with large-scale environmental gradients (100–1000 km), and secondarily with meso-scale gradients (10–100 km). This study increases our understanding of the Arctic that is experiencing rapid changes and could serve as a benchmark against which future changes in megabenthic diversity and community patterns could be identified (e.g., species range shifts, invasive species).

## Materials and Methods

### 2.1. Study Area

**Figure 1:**
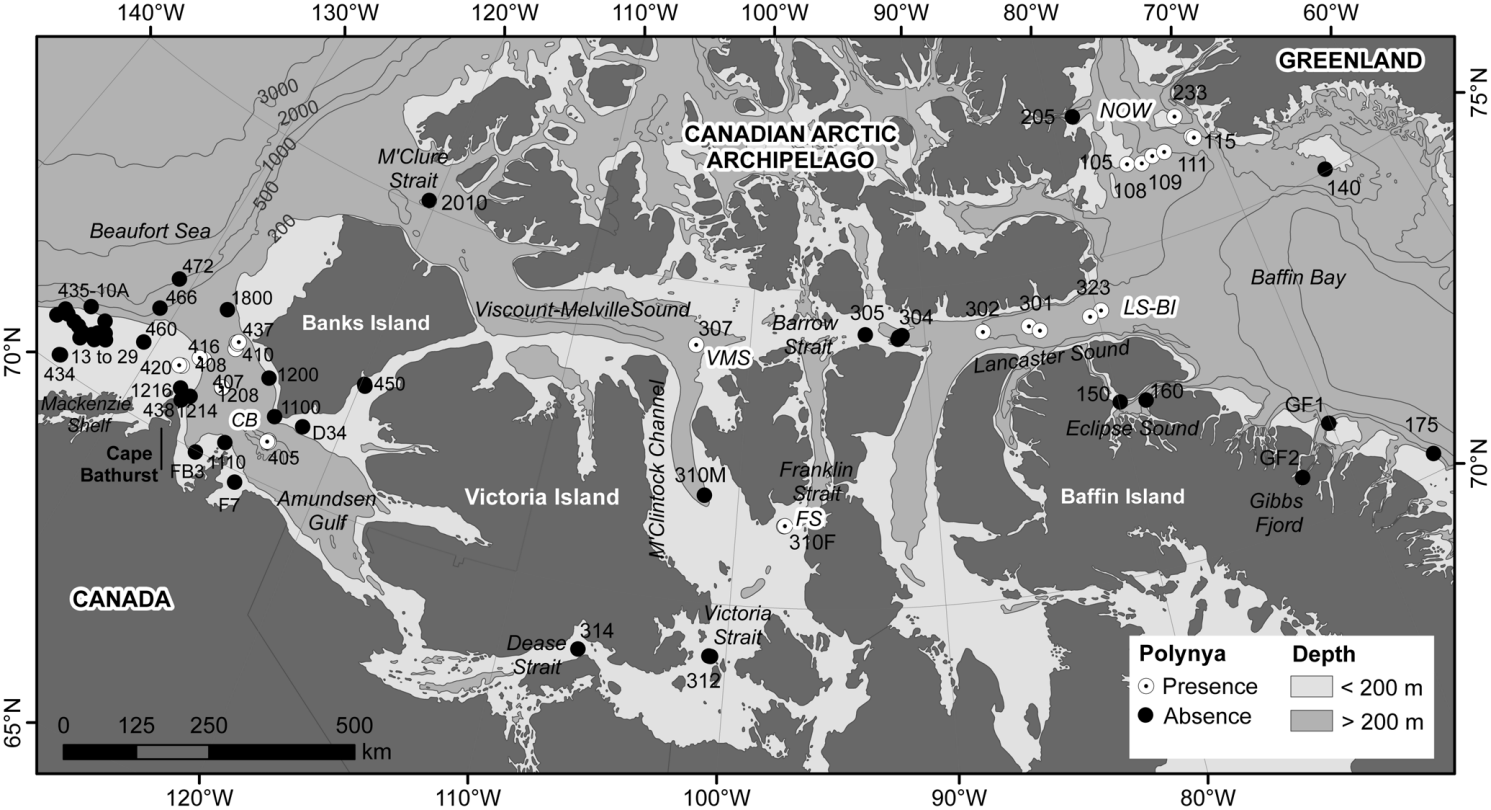
Locations of stations sampled from 2007 to 2011 across the Canadian Arctic. Stations sampled in areas where polynyas are recurrently present (white circles) or absent (black circles). Station codes correspond to ArcticNet expedition labels, sampling years were not added for clarity (see Table S2 in File S1). Names of polynyas are indicated by capital italic letters (*CB*: Cape Bathurst polynya, *FS*: Franklin Strait polynya, *LS-BI*: Lancaster Sound-Bylot Island polynya, *NOW*: North Water polynya, *VMS*: Viscount-Melville Sound polynya). The shelf break and the transition between the Pacific and Atlantic water masses are both around 200 m (<200 m: shelf and Pacific layer; >200 m: slope and Atlantic layer).

This study was conducted across the Canadian Arctic from the Mackenzie Shelf in the southeastern Beaufort Sea in the west (135°W) to northern Baffin Bay in the east (65°W) (Figure 1). The two main water masses flowing through the Canadian Arctic originate mainly from the Pacific and Atlantic oceans. The colder-fresher Pacific-origin waters (on average<200 m depth) overlie the warmer-saline Atlantic-origin waters below (on average>200 m depth) [26]. The transition between these water masses coincides generally across the study area with the 200 m isobath along the shelf break [28], [29]. The Beaufort Sea and Amundsen Gulf regions are highly influenced by the Mackenzie River that drains a watershed of 1.7×10^6^ km^2^ and discharges approximately 340 km^3^ y^−1^ of freshwater [26] and 127×10^6^ Mt y^−1^ of sediment load [30] into the Beaufort Sea. The complex topography of the Canadian Arctic Archipelago with its numerous islands and channels has a profound influence on sea ice circulation and marine biological productivity regimes [31]. During winter the study area is ice-covered and sea ice could be found throughout the summer as landfast ice or first-year and multiyear pack ice [32], [33]. Summer sea ice distribution along with ice break-up and freeze-up dates exhibit large inter-annual variations [32], [33]. As a general trend, ice in summer remains longer in the central part of the Archipelago than in areas where large and latent heat polynyas open in spring, such as the North Water (NOW), Lancaster Sound-Bylot Island (LS-BI), and the Cape Bathurst (CB) polynyas [31], [34] (Figure 1). Polynyas located in the northeastern Canadian Arctic (i.e., NOW and LS-BI) exhibit intense marine biological productivity and tight pelagic-benthic coupling as revealed by field observations of diatom-based communities [27], satellite-derived high annual primary production (PP) estimates [35], and high sediment chlorophyll *a* (Chl *a*) concentrations and benthic boundary fluxes [36], [37]. In the CB polynya, in contrast, highly variable intensity, timing and duration of phytoplankton blooms [38], and strong grazing pressure by zooplankton leads to weak pelagic-benthic coupling [37], [39], [40]. The central Archipelago has been defined as an oligotrophic system [27].

### 2.2. Ethics Statement

Sampling licenses were obtained for the Northwest Territories (Canada) by the Aurora Research Institute (#14258, #14304, #14543, #14678, #14917), by the Environmental Impact Screening Committee (#06 07 05, #06 03 10, #03 09 03), and by the Department of Fisheries and Oceans (DFO) (#S-07/08-4017-IN, #S-09/10-4013-IN, #S-10/11-3026-YK, #S-11/12-3026-YK). For Nunavut (Canada) permits were provided by the Nunavut Research Institute (#0500907R-M, #0501408R-M, #0504609R-M, #0505510R-M, #0506511R-M) and by DFO (S-07/08-1034-NU, #S-08/09-1043-NU, # S-09/10-1049-NU, #S-10/11-1021-NU, #S-11/12-1029-NU).

### 2.3. Faunal data collection

Benthic fauna were sampled at 78 stations between June and October from 2007 to 2011 onboard the Canadian research icebreaker CCGS *Amundsen* (Table S1 in File S1). Station depths ranged from 34 to 1024 m, all below the average ice scouring zone [28], [41]. All faunal samples were collected with an Agassiz trawl (effective opening of 1.5 m and a 40 mm net mesh size, with a 5 mm cod end liner) with average trawling time and speed of 5 min and 1.5 knots, respectively. In order to standardize community characteristics among stations (by m^−2^), bottom trawling time and vessel speed at each station were used to calculate towed area (trawl opening of 1.5 m× distance towed; average trawled area of 372±161 m^−2^). This trawl design is very effective at collecting both epibenthic and burrowing, large-sized invertebrates. Based on the methodology of Piepenburg et al. [19], invertebrates larger than 2 cm were sorted from the trawl catches directly after capture and classified as megabenthos. In addition, the sediment contained in the catches was washed through a 2 mm sieve under running seawater onboard [19]. Planktonic invertebrates that were accidentally taken by the trawl (e.g., Chaetognatha, Euphausiacea, Scyphozoa) and Pisces were removed to only include benthic invertebrates in the sample analysis. Members of the class Ascidiacea were not considered in this study due to exclusion of this taxon during the first years of sampling. Only large echinoderm taxa that could be reliably identified to species level were counted and wet-weighed in the field given the low precision of on-board mass measurements (detection limit of 5 g). All other taxa were preserved in a 4% seawater-formaldehyde solution buffered with sodium tetraethylborate or frozen for later identification in the lab, and their biomass was determined as formaldehyde wet mass or wet mass (after thawing) at 0.001 g precision. Possible biases in total biomass calculations introduced by different preservation methods were considered minor since all specimens within a phylum were processed the same way and trawl catches were considered semi-quantitative estimates [42], [43]. Only specimens with the head-part intact were counted and identified to the lowest possible taxonomic level. However, some taxa were left at the phylum level because no complete identification keys exist for Canadian Arctic waters (e.g., Brachiopoda, Nemertea, Platyhelminthes, Porifera); we acknowledge that their richness will have been underestimated in this study. Taxonomic names were verified using the World Register of Marine Species (WoRMS, [44]). Four species of the phylum Bryozoa and one from the phylum Hydrozoa were not listed in WoRMS but were verified using the Integrated Taxonomic Information System (ITIS, www.itis.gov) (i.e., Bryozoa: *Cellepora smitti, Escharopsis rosacea, Escharopsis sarsi, Porella sacata*; Hydrozoa: *Obelia loveni*).

### 2.4. Environmental data collection

Explanatory environmental variables available for the present study (Table S2 in File S1) were divided into three categories: resource, direct and indirect/spatial gradients (following McArthur et al. [14]). Resource gradients included estimates of vertical POC fluxes derived from primary productivity in surface waters (e.g., phytoplankton biomass, PP estimates) to sediment variables that were proxies of the energy available for benthic consumers (e.g., sediment pigments, sediment organic carbon). Resource gradient variables are called hereafter ‘food supply proxies'. Direct gradients included bottom oceanographic variables (i.e., temperature, oxygen, salinity), seabed substrate type (hard vs. soft) and terrestrial influence on the benthic habitat (i.e., sediment δ^13^C), these variables selecting for the type of physiology, morphology and/or life history of species residing there. Finally, indirect/spatial gradients consisted of purely spatial variables (depth, latitude and longitude) that often correlate with direct and resource variables but with no direct physiological influence on the species. All these environmental gradient categories vary on different temporal scales and we assessed their temporal variability as follow (Table 1). Spatial variables were assumed temporally stable, except on geological time scales. Direct variables were overall assumed to be unchanging on less than a decadal scale [31]. Food supply proxies fluctuate either on a seasonal basis (e.g., phytoplankton biomass [27] and sediment Chl *a* [45], [46]) or on a multi-annual basis (e.g., PP estimates integrated over years, sediment organic carbon [47], [48]). Additionally, these environmental categories exhibit measurable heterogeneity at different spatial scales, from study area extent to distance between stations. In a continental-scale study such as this one, indirect/spatial and direct gradients should mostly influence benthic community patterns over large geographic scales (100–1000 km), while resource gradients should induce environmental heterogeneity mainly at meso-geographic scales (10–100 km) (Table 1) [13].

**Table 1:** Spearman rank correlation coefficients for relationships between benthic univariate community characteristics and environmental variables. Significant correlations (*p*<0.01) are indicated in bold.

Spatial variability	Meso to large scale (in continental-scale study)						Large scale (100–1000 km)							Meso scale (10–100 km)							
Temporal variability	Years to decades						Relatively stable			Low (>10 years)			Medium (1–10 years)				High (seasonal)				
Variable type	Benthic community characteristic						Indirect/spatial gradient			Direct gradient				Resource gradient/food supply proxy							
	Biomass	Density	S_density_	H′	J′	Δ*	Latitude	Longitude	Depth	Temperature	Salinity	Oxygen	Sed. δ^13^C	Sed. OC	PP 5Y	PP 1Y	Sed. phaeo	Sed. Chl *a*	Euphotic B_T_	Euphotic B_S_	Euphotic B_L_
Biomass	1.00																				
Density	**0.84**	1.00																			
S_density_	**0.64**	**0.73**	1.00																		
H′	−0.05	0.20	**0.30**	1.00																	
J′	**−0.40**	−0.15	−0.20	**0.79**	1.00																
Δ*	−0.11	−0.07	0.00	−0.12	−0.05	1.00															
Latitude	0.07	−0.11	−0.11	**−0.44**	**−0.35**	−0.06	1.00														
Longitude	0.01	−0.16	−0.07	−0.27	−0.16	0.04	**0.57**	1.00													
Depth	−0.25	**−0.34**	−0.26	**−0.39**	−0.20	−0.02	**0.66**	**0.65**	1.00												
Temperature	−0.12	−0.19	−0.18	**−0.37**	−0.19	−0.11	**0.46**	**0.46**	**0.76**	1.00											
Salinity	**−0.38**	**−0.44**	**−0.51**	**−0.45**	−0.11	−0.04	**0.47**	0.29	**0.79**	**0.71**	1.00										
Oxygen	0.22	**0.30**	**0.43**	**0.38**	0.08	0.02	**−0.50**	**−0.55**	**−0.67**	**−0.61**	**−0.65**	1.00									
Sed. δ^13^C	−0.06	−0.25	−0.25	−0.28	−0.20	0.03	**0.59**	**0.76**	**0.60**	**0.37**	**0.37**	**−0.55**	1.00								
Sed. OC	0.01	0.00	−0.02	0.15	0.12	−0.01	0.26	−0.09	0.16	0.12	0.15	−0.17	0.06	1.00							
PP 5Y	**0.45**	**0.38**	0.26	0.16	−0.01	−0.10	−0.07	−0.30	nr	nr	nr	nr	−0.31	**0.57**	1.00						
PP 1Y	**0.44**	**0.35**	0.30	0.20	−0.01	−0.22	−0.07	−0.30	nr	nr	nr	nr	−0.29	**0.51**	**0.92**	1.00					
Sed. phaeo	0.25	0.17	0.09	0.17	0.05	0.02	0.24	0.10	−0.01	−0.08	−0.19	−0.06	0.17	**0.65**	**0.56**	**0.44**	1.00				
Sed. Chl *a*	**0.36**	0.31	0.25	0.28	0.05	−0.08	−0.03	−0.15	**−0.38**	−0.33	**−0.54**	0.28	0.03	**0.51**	**0.58**	**0.55**	**0.80**	1.00			
Euphotic B_T_	−0.18	−0.21	−0.15	−0.20	−0.15	−0.04	0.17	**0.45**	nr	nr	nr	nr	**0.61**	−0.18	**−0.35**	**−0.40**	−0.03	−0.12	1.00		
Euphotic B_S_	−0.12	−0.07	0.06	−0.04	−0.08	−0.06	−0.12	0.05	nr	nr	nr	nr	−0.10	−0.06	−0.24	−0.31	−0.21	−0.31	**0.55**	1.00	
Euphotic B_L_	0.18	0.13	0.10	−0.03	−0.19	−0.12	0.13	0.24	nr	nr	nr	nr	**0.55**	0.13	0.07	−0.03	**0.36**	**0.40**	**0.89**	0.21	1.00
Euphotic B_L_:B_T_	0.28	0.25	0.04	0.06	−0.01	−0.08	0.05	0.10	nr	nr	nr	nr	**0.50**	0.19	0.30	0.24	**0.47**	**0.56**	**0.45**	−0.34	**0.78**

Benthic community characteristics: biomass (g m^−2^); density (ind. m^−2^; without colonial organisms); S_density_: taxon richness density (number of taxa m^−2^); H′: Shannon–Wiener's diversity index; J′: Pielou's evenness index; Δ*: average taxonomic distinctness. Indirect/spatial gradients: latitude and longitude (km; starting at the most southwestern station); depth (m). Direct gradients: Bottom oceanographic variables: temperature (°C); salinity; oxygen (ml l^−1^); Terrestrial influence: sediment δ^13^C (‰). Resource gradients/food supply proxies: sed. OC: sediment organic carbon (%); PP: sum of monthly satellite-derived primary production estimates over one (PP 1Y) or five years (PP 5Y) before sampling (mg C m^−2^ y^−1^; model results of Bélanger et al. [35]); sed. phaeo: sediment phaeopigments (µg g^−1^); sediment Chl *a* (µg g^−1^); euphotic B_T_: total phytoplankton biomass (cells ≥ 0.7 µm; mg Chl *a* m^−2^); euphotic B_S_: biomass of small phytoplankton cells (0.7−5 µm; mg Chl *a* m^−2^); euphotic B_L_: biomass of large phytoplankton cells (≥ 5 µm; mg Chl *a* m^−2^); euphotic B_L_:B_T_: relative contribution of large cells to total biomass. nr: biologically not relevant.

#### 2.4.1. Food supply proxies - primary productivity

We used pelagic primary productivity estimates as food supply proxies for benthic organisms based on the assumption that areas with higher pelagic primary productivity should generally have higher vertical POC fluxes [14]. We consequently evaluated if the spatial variability in primary productivity of surface waters was linked to the spatial variability observed in benthic community patterns. Various estimates of primary productivity differ in their temporal integration of the variability of a system. For seasonal variability, we used phytoplankton biomass estimates based on water Chl *a* concentrations measured at the time and locations of faunal sampling and integrated over the euphotic zone (from surface to 0.2% surface light level). We also tested if different size fractions of phytoplankton biomass estimates would be linked with the same strength to benthic community patterns, as large cells sink rapidly and are therefore supposed to contribute most to the carbon flux reaching the seafloor [49]. We estimated the following phytoplankton biomass size fractions: euphotic BT  =  total phytoplankton biomass (cells ≥ 0.7 µm; mg Chl *a* m^−2^); euphotic BS  =  biomass of small phytoplankton cells (0.7−5 µm; mg Chl *a* m^−2^); euphotic BL  =  biomass of large phytoplankton cells (≥ 5 µm; mg Chl *a* m^−2^); and euphotic BL:BT  =  relative contribution of large cells to total biomass (Table 1). Data were available at 73 stations and details on the sampling and analytical methods are found in Ardyna et al. [27]. In addition, we summed satellite-derived monthly PP estimates to assess annual variability of primary productivity (Table 1). Sums of monthly PP estimates over one (PP 1Y) and five years (PP 5Y) before faunal sampling were determined for a 20 km radius around each sampling station based on model results of Bélanger et al. [35] (data available for 71 stations). Sampling stations were also categorized according to presence (n = 30 stations) and absence (n = 48 stations) of a polynya (based on Arrigo and van Dijken [38] and Barber and Massom [50]) as a proxy of ice conditions and primary productivity.

#### 2.4.2. Food supply proxies - surface sediment

We evaluated the seasonal contribution of ‘fresh’ organic matter inputs to the benthos as sediment Chl *a* and phaeopigments (degraded chlorophyll) concentrations, and by using sediment organic carbon as an estimate of average annual input. From 2008 to 2011, a USNEL box corer (0.25 m^2^) was deployed for collecting surface sediments (upper 1 cm) in triplicate using a 60 ml disposable syringe (2.6 cm diameter with a cut off anterior end). Sediment samples for pigment concentration (Chl *a* and phaeopigments) and organic carbon content were immediately frozen at −80°C and −20°C, respectively, for later analysis in the lab. Pigment concentrations were analysed fluorometrically following a modified protocol by Riaux-Gobin and Klein [51] and are expressed as microgram pigment per gram of dry sediment. Sediment organic carbon content was determined after acidification (HCl 10%) with a Costech 4010 elemental analyser (Marine Chemistry and Mass Spectrometry Laboratory, Université du Québec à Rimouski, Canada). Sediment organic carbon content is expressed as % of total sediment dry weight.

#### 2.4.3. Terrestrial organic matter input

Sediment δ^13^C was used as a measure of the contribution of terrestrial organic carbon input in order to investigate influence of coastal erosion and river sediment discharge on the benthic community structure. Sediment samples were collected and preserved the same way as sediment organic carbon described above. Sediment δ^13^C was determined after acidification (HCl 10%) with a CF-IRMS (continuous-flow Isotope Ratio Mass Spectrometry) (Marine Chemistry and Mass Spectrometry Laboratory, Université du Québec à Rimouski, Rimouski, Québec, Canada) and is reported in standard delta notation in ‰ with respect to VPDB (Vienna Pee Dee Belemnite). Lighter sediment isotopic δ^13^C values (−28 to −26‰) are typical of terrigenous organic matter while heavier isotopic δ^13^C values (−24 to −20‰) are typical of marine production [52].

#### 2.4.4. Bottom oceanographic variables

Bottom water characteristics were measured at all stations from 2007 to 2011. Near-bottom water temperature (°C), salinity and dissolved oxygen concentration (ml l^−1^) were determined by the shipboard CTD Seabird profiler (SBE911 Plus), combined with a SBE 43 dissolved oxygen sensor, at 10 m above the seafloor.

#### 2.4.5. Substrate type

Because sediment particle size samples could not be consistently sampled during all years, we instead used a qualitative classification based on visual observations of trawls and box corers to assess the substrate type at each station. Substrate category ‘hard’ was assigned to stations with substantial amounts of gravel and cobbles, and ‘soft’ assigned to stations with mud (silt and clay), sand and no or little gravel. Overall, fewer hard substrate stations (19 of 78 total stations) were sampled to avoid damaging the trawl and box corer, so that hard bottom stations are under-represented in this study. Near-bottom current speed could not be assessed for this study, but substrate type may be regarded as a proxy for current velocity with coarser substrate indicating a higher near-bottom flow regime [25].

### 2.5. Data analysis

Benthic community characteristics considered in this study for each of the 78 stations were biomass (g m^−2^), density (ind. m^−2^), and four biodiversity metrics (taxonomic richness density (S_density_, number of taxa 1000 m^−2^), Shannon-Wiener's diversity index (H′, using log_e_), Pielou's evenness index (J′), and average taxonomic distinctness (Δ*)). H′, J′ and Δ* were calculated based on biomass data including colonial taxa. Density was calculated after removal of colonial taxa because their abundance cannot be recorded (i.e., Bryozoa, Hydrozoa, Nephtheidae (soft corals), Porifera). Δ* estimates the average distance between two randomly chosen organisms through Linnean taxonomy and is considered to be a more genuine reflection of biodiversity than the other diversity indices because it considers taxonomic relationships [53]. Six taxonomic levels were used in Δ* calculations: species, genus, family, order, class and phylum, assuming equal step weights between successive taxonomic levels; when necessary, the lowest taxonomic level available was used for missing level(s) (performed using PRIMER-E software version 6 [54]). Correlations between benthic community characteristics and quantitative environmental variables were assessed using Spearman rank correlations to investigate the intensity of all possible relations following a positive or negative monotonic trend [55]. Prior to correlation analysis, we verified by visual observation that no relationship was quadratic (hump-shape curve). Simple linear regressions were performed to model the relationships between benthic community characteristics and depth as an environmental proxy measure often used in benthic studies. Normality of residuals was examined by plotting theoretical quantiles vs. standardized residuals (Q-Q plots) and homogeneity of variance was assessed by plotting residual vs. fitted (predicted) values. Mann-Whitney *U* tests were used to seek differences in benthic community characteristics between the environmental categories substrate type (hard vs. soft) and polynya (presence vs. absence). Kruskal-Wallis tests with post-hoc multiple comparison tests were carried out to test differences among community clusters (see below).

For multivariate analyses, lists of taxa at each station were scaled at the genus level and taxa only found at one station were discarded, for a total of 303 unique taxa found at least at two stations. Singletons in multivariate analysis are prone to random and uninterpretable fluctuations, and it is consequently suggested to remove them to allow better detection of the underlying community similarities [53]. Scaling at the genus level was done because identifications were patchy at the species level among stations; in some cases, specimens were incomplete and missing criteria prevented identification at the species level. Bray-Curtis dissimilarity was calculated for the fourth-root-transformed biomass matrix rather than for the density matrix to be able to include colonial taxa. The fourth-root transformation was chosen to balance the effects of high- and low-biomass taxa to assess responses of the whole communities [53]. The dissimilarity matrix was then subjected to a hierarchical cluster analysis using Ward's minimum variance method, which seeks to define well-delimited groups by minimizing within-cluster sum of squares [56]. Community clusters were determined by selecting a distance where stations were fused in well-defined clusters. To find indicator taxa within each community cluster, the indicator value index (IndVal) method of Dufrêne and Legendre [57] was applied on the biomass data matrix. IndVal is a measure of association between a taxon and a cluster of stations and is calculated as the product of specificity (mean biomass of a given taxon within a cluster compared to the other clusters) and fidelity (taxon occurrence at stations belonging to a cluster). IndVal is maximal ( = 100%) when a given taxon is observed at all stations of only one community cluster and in none of the other clusters. Statistical significances of indicator taxa were tested by random permutation of stations (9999 permutations) and only the five significant indicator taxa with the greatest IndVal value are discussed per community cluster. The influence of all environmental variables on the taxonomic composition was tested on 50 stations (out of 78 stations total) by the use of redundancy analysis (RDA), a direct extension of regression analysis to model multivariate response data. The other 28 stations had to be removed (2007: all 10 stations; 2008: n = 9; 2009: n = 1; 2010 n = 3: 2011: n = 5) because of some missing food supply proxies. A Principal Component Analysis (PCA) plot showing the multivariate similarity among the 50 stations in terms of environmental variables is available in Supporting Information (Figure S1 in File S1). Removing stations for the RDA reduced the total number of taxa found at least at two stations from 303 to 266. The RDA was performed after Hellinger transformation to reduce the importance of dominant taxa [58]. Environmental variables entered into the model were: seven food supply proxies (polynya presence/absence, PP 1Y and PP 5Y, sediment organic carbon, sediment phaeopigments, sediment Chl *a*, and euphotic B_T_), five direct variables (three bottom oceanographic variables (bottom oxygen, salinity and temperature), substrate type, sediment δ^13^C), and three indirect/spatial variables (depth, latitude, longitude). We performed two RDA: one included variables from all types of environmental categories and the other excluded indirect/spatial variables because the latter may mask food supply and direct gradients that have higher ecological significance [14]. Reduction of explanatory variables was performed by forward selection on the basis of their permutation *p* values (9999 permutations) and on Akaike's Information Criterion (AIC) in case of ties. Collinearity of significant forward selected explanatory variables was verified looking at variance inflation factors (VIF)<10 [55].

Statistical analyses were performed using the statistical package R version 3.0 [59]. Statistical significance at *α*<0.05 was used for all statistical tests except for Spearman correlations and Kruskal-Wallis post-hoc multiple comparison tests, where a statistical significance at *α*<0.01 was used to account for the increasing probability of type I error in multiple testing [55]. The distribution of biomass, density, S_density_ and H′ were mapped with ArcGIS 9.3.1 with color bins defined by the Jenks iterative method which minimizes within class difference and maximizes between class differences [60].

## Results

### 3.1. Community characteristics: biological and environmental linkages

**Figure 2:**
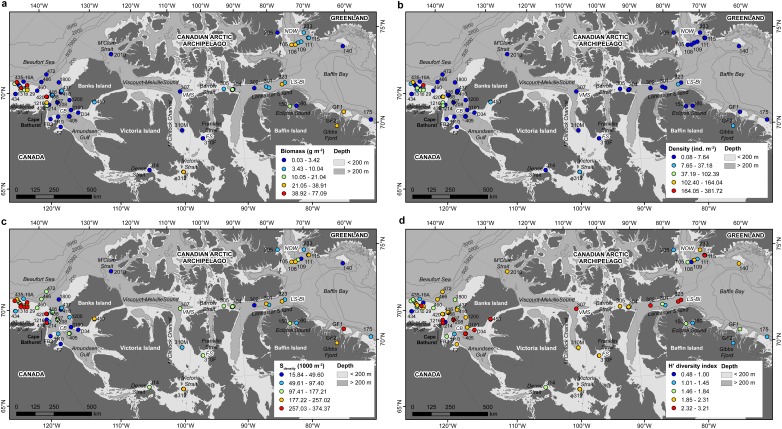
Distributions of benthic community characteristics at 78 stations over 2007–2011. (**a**) biomass (g m^−2^); (**b**) density (ind. m^−2^); (**c**) S_density_ (no. of taxa 1000 m^−2^); (**d**) Shannon-Wiener's diversity index (H′).

A total of 527 unique taxa were identified at the lowest possible taxonomic level across all 78 stations (430 at the species level) (Table S3 in File S1). Faunal biomass across all stations ranged from<1 to 77 g m^−2^, density from<1 to 382 ind. m^−2^, S_density_ from 16 to 374 taxa 1000 m^−2^, H′ from 0.48 to 3.21, J′ from 0.16 to 0.85, and Δ* from 71.8 to 99.4 (Table S1 in File S1). Distribution of benthic biomass, density, S_density_ and H′ showed some distinct spatial patterns (Figure 2); J′ and Δ* were not mapped due to their poor association with environmental gradients. Density, biomass and S_density_ were positively correlated with each other, as were H′ and J′ (Table 1). Biomass and J′ were negatively correlated, and Δ* was not correlated with any community characteristics and also with no environmental variables.

**Figure 3:**
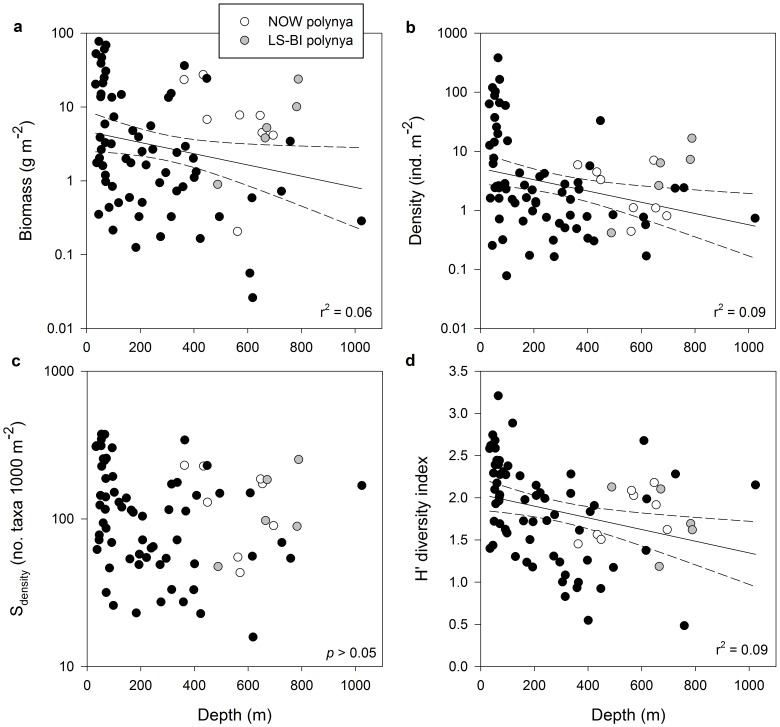
Relationships of benthic community characteristics with depth. Stations sampled underneath Lancaster Sound-Bylot Island polynya (LS-BI; gray circles) and NOW polynya (white circles) are highlighted. (**a**) biomass (g m^−2^); (**b**) density (ind. m^−2^); (**c**) S_density_ (no. of taxa 1000 m^−2^); (**d**) Shannon-Wiener's diversity index (H'). Coefficients of determination of significant linear regressions (*p*<0.05) are shown and dashed lines represent 95% confidence intervals.

Among the relationships tested with indirect/spatial variables, H′ and J′ were negatively correlated with latitude (from south to north); density and H′ were negatively correlated with depth. Regression models relating benthic biomass, density, S_density_ and H′ with depth had poor explanatory power, in part due to the positive influence of the productive LS-BI and NOW polynyas at deep stations (Figure 3). Among the correlations tested with bottom oceanographic variables, H′ was negatively correlated with temperature; biomass, density, S_density_ and H′ were negatively correlated with salinity; density, S_density_ and H′ were positively correlated with oxygen. Among the correlations tested with food supply proxies, biomass and density were positively correlated with PP 1Y and PP 5Y, and biomass was positively correlated with sediment Chl *a* (Table 1). No benthic community characteristic was significantly correlated with sediment δ^13^C, sediment organic carbon, sediment phaeopigments, or any descriptors of euphotic phytoplankton biomass. Lower S_density_ and H′ values were found in hard substrate stations than in soft substrate stations (Table 2). H′ was significantly lower at stations located within than outside a polynya (Table 2).

**Table 2:** Results of significant differences in benthic community characteristics between environmental categories and among community clusters.

Community characteristic/Categorical variable	Substrate type (Hard vs. Soft)	Polynya (Presence vs. Absence)	Community clusters (significant differences are shown)
Biomass	ns	ns	Deep coldspots < Local hotspots; Shelf break < Local hotspots; Deep coldspots < Mackenzie Shelf; Shelf break < Mackenzie Shelf
Density	ns	ns	Deep coldspots < Local hotspots; Deep coldspots < Mackenzie Shelf; Shelf break < Mackenzie Shelf
S_density_	Hard < Soft	ns	Deep coldspots < Mackenzie Shelf; Shelf break < Mackenzie Shelf
H′	Hard < Soft	Presence < Absence	Deep coldspots < Mackenzie Shelf; Local hotspots < Mackenzie Shelf
J′	ns	ns	ns
Δ*	ns	ns	ns

Mann-Whitney *U* tests were applied on categorical variables with two states, while Kruskal-Wallis tests were used to test for difference among community clusters (post-hoc comparisons at α = 0.01). ns: non-significant.

Spatial variables were highly correlated (correlation coefficient>0.5) with all direct gradient variables (bottom oceanographic variables and sediment δ^13^C) (Table 1). Sediment Chl *a* was the only sediment food supply proxy correlated negatively with depth. Food supply proxies in surface waters representing different temporal integration of primary productivity varied in opposite directions: integrated PP estimates (PP 1Y and PP 5Y) and seasonal euphotic B_T_ were negatively correlated. However, food supply proxies in sediment varied in the same direction: sediment organic carbon, sediment phaeopigments and sediment Chl *a* were positively correlated. These latter three sediment food supply proxies also were positively correlated with PP 1Y and PP 5Y; only sediment phaeopigments and sediment Chl *a* were positively correlated with the highly seasonal euphotic B_L_ and euphotic B_L_:B_T_ (Table 1).

### 3.2. Community clusters and distribution patterns

**Figure 4:**
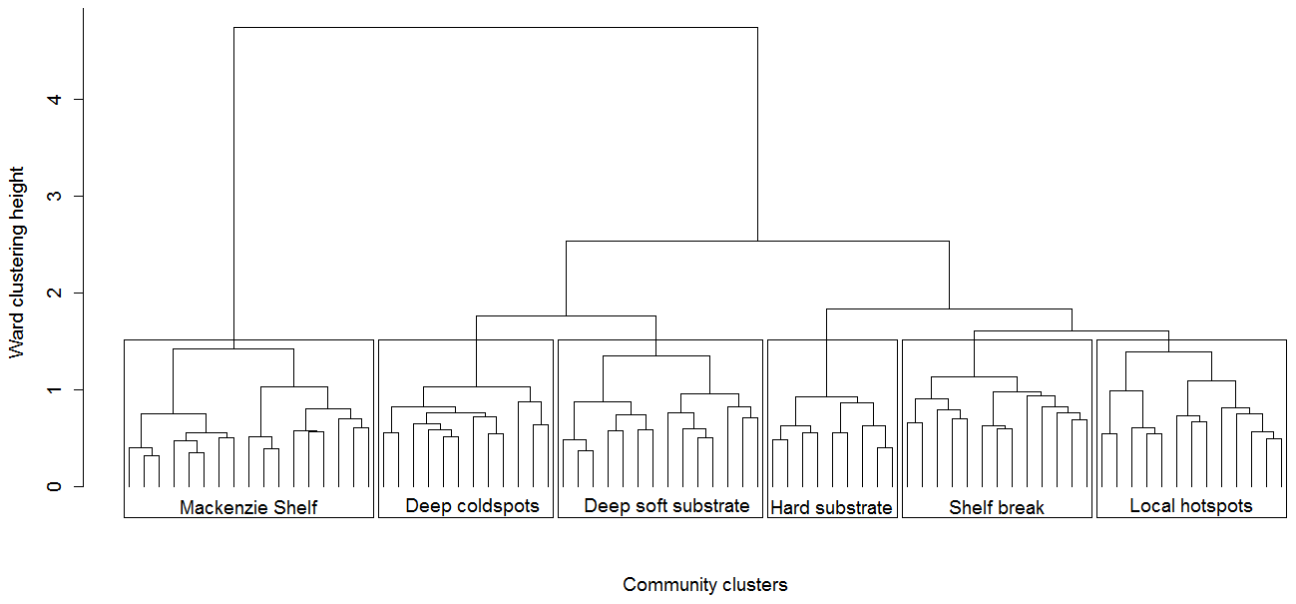
Community cluster partition. Ward's minimum variance cluster analysis based on Bray-Curtis dissimilarity matrix using fourth-root transformed megafaunal biomass data at 78 stations over 2007–2011.

**Figure 5:**
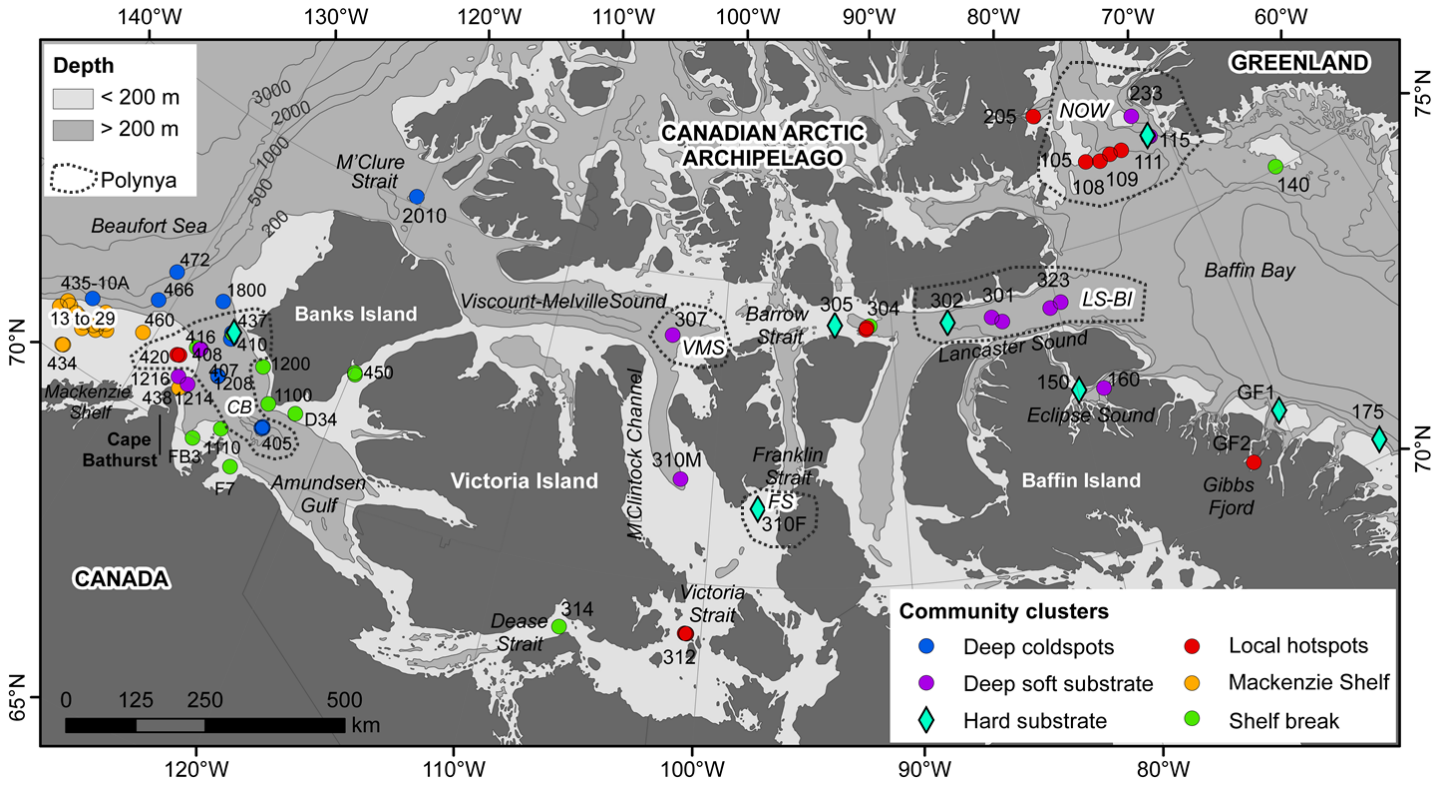
Locations of the six megabenthic community clusters.

**Figure 6:**
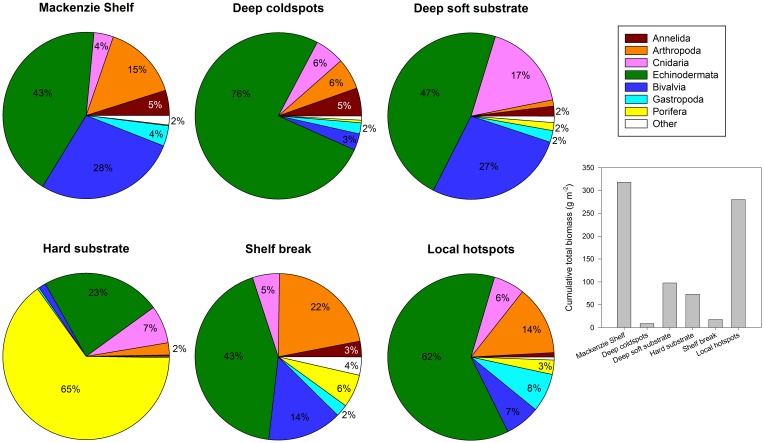
Variation in mean relative biomass composition (%; only≥2% shown) for the main phyla or classes sampled in all community clusters (pie charts) and cumulative total biomass (g m^−2^) sampled per community cluster (histogram).

Ward clustering analysis resulted in six well-defined community clusters (Figure 4). We attributed a ‘label’ to each community cluster based on three variables (mean biomass, mean depth, proportion of hard/soft substrate stations) and their respective minimal and maximal values among clusters (Table 3). The term ‘coldspots’ was attributed to the community cluster with the lowest mean biomass, and the term ‘hotspots’ was given to the community cluster characterized by highest mean biomass. Because the ‘hotspots’ community type was spatially distributed at discrete locations across the study area, we named it ‘local hotspots’ community. Substrate type (hard or soft) was added to the name of a cluster when almost all, if not all stations, were of one substrate type. The mean depth around the 200 m shelf break was chosen as the main attribute for the ‘shelf break’ cluster. Depth was used as a descriptor when all stations were deeper than 200 m (only station 1216 in ‘deep soft substrate’ cluster was <200 m, Figure 5). The ‘Mackenzie Shelf’ community cluster was the only one named based on its geographical location (Figure 5) and was the most dissimilar in terms of taxonomic composition compared with all other clusters (Figure 4). The other five community clusters formed two major groups: one group with two clusters found at deep stations (‘deep coldspots’ and ‘deep soft substrate’ clusters) and the second group composed of the remaining three community clusters (‘hard substrate’, ‘shelf break’, ‘local hotspots’) (Figure 4). Across all community clusters, dominant taxa in terms of biomass were often large echinoderms (e.g., sea star, brittle star, basket star, sea cucumber), sea anemones and sponges, but also high biomass of the bivalves *Astarte* spp. and isopods *Saduria* spp. prevailed in some community clusters (Table 3). The distinctiveness of the ‘Mackenzie Shelf’ taxonomic composition was well represented by the high ‘IndVal’ index values (≥82%) of the top five significant indicator taxa, meaning that those taxa were almost exclusively found in this community cluster (Table 3). Comparatively, the significant indicator taxa of the other five community clusters had ‘IndVal’ values between 22% and 62% and occurred in more than one community cluster (Table 3). The ‘Mackenzie Shelf’ community cluster was composed of stations with significantly higher biomass, density and S_density_ than stations in ‘deep coldspots’ and ‘shelf break’ clusters, and with higher H′ than ‘deep coldspots’ and ‘local hotspots’ communities (Table 2). ‘Local hotspots’ community cluster stations had greater biomass than stations in ‘deep coldspots’ and ‘shelf break’ communities, and greater density than ‘deep coldspots’ cluster stations (Table 2). J′ and Δ* were not significantly different among community clusters (Table 2). Relative mean biomass contribution of the main phyla and cumulative total biomass varied between community clusters (Figure 6). The ‘Mackenzie Shelf’ community cluster was characterized by high biomass of Echinodermata (43%) and Bivalvia (28%). Echinodermata dominated biomass (76%; mostly Ophiuroidea) at almost all stations of the ‘deep coldspots’ cluster. Biomass of Echinodermata (47%; almost equally Asteroidea, Crinoidea, Holothuroidea and Ophiuroidea), Bivalvia (27%) and Cnidaria (17%; mostly Anthozoa) were high at several stations of the ‘deep soft substrate’ cluster. For stations of the ‘hard substrate’ cluster, Porifera (65%) and Echinodermata (23%; mostly Echinoidea) were dominating biomass. ‘Shelf break’ and ‘local hotspots’ clusters were similar in relative biomass with high Echinodermata biomass (43–62%; both having predominantly high biomass of Echinoidea, but successively high biomass of Holothuroidea for ‘local hotspots’ and high biomass of Asteroidea and Ophiuroidea for ‘shelf break’), and high biomass of Arthropoda (14–22%) and Mollusca (15–16%). A station-based account of the relative contribution of the main phyla for biomass and taxonomic richness is available in the Supporting Information (Figure S2 in File S1).

**Table 3:** Megabenthic community clusters and their respective total taxonomic richness, mean biomass, mean depth, as well as the proportion of hard/soft substrate stations in each cluster (variables used to attribute a ‘label’ to each community are in bold).

Community cluster	No. of stations N = 78	Total taxonomic richness (range for stations)	Mean biomass (± SD) (g m^−2^)	Mean depth (± SD) (m)	Substrate type proportion (hard/soft stations)	Dominant taxa	Fidelity (%)	Significant indicator taxa -ex aequo are shown-	Specificity (%)	Fidelity (%)	IndVal (%)
Mackenzie Shelf	17	179 (25–119)	18.70 (21.85)	58 (14)	0/17	*Icasterias* sp. (Sea star)	53	*Cistenides* sp.(Polychaete: Pectinariidae)	100	100	100
						*Astarte* spp. (Bivalve)	65	*Macoma* spp. (Bivalve)	99	100	99
						*Saduria* spp. (Isopod)	100	*Saduria* spp. (Isopod)	93	100	93
						*Gorgonocephalus* spp. (Basket star)	29	*Pontoporeia* sp. (Amphipod)	100	88	88
						*Ophiocten* sp. (Brittle star)	82	*Ciliatocardium* sp. (Bivalve)	93	88	82
Deep coldspots	12	114 (11–52)	**0.73 (0.61)**	**498 (199)**	3/9	*Ophiopleura* sp. (Brittle star)	92	*Bythocaris* sp. (Decapod)	56	75	42
						*Ophiacantha* sp. (Brittle star)	67	*Amage* sp. (Polychaete: Ampharetidae)	96	33	32
						*Pontaster* sp. (Sea star)	67	*Siphonodentalium* sp. (Scaphopod)	50	58	29
						*Ctenodiscus* sp. (Sea star)	17	*Apherusa* sp. (Amphipod), *Sarsiflustra* sp. (Bryozoan)	100	25	25
						*Icasterias* sp. (Sea star)	25	*Myrioglobula* sp. (Polychaete: Oweniidae)	98	25	24
Deep soft substrate	14	126 (11–64)	6.96 (9.09)	**485 (261)**	**1/13**	*Gorgonocephalus* spp. (Basket star)	14	*Pontaster* sp. (Sea star)	60	79	47
						*Astarte* spp. (Bivalve)	100	*Psilaster* sp. (Sea star)	100	43	43
						*Actinauge* sp. (Anemone)	29	*Asychis* sp. (Polychaete: Maldanidae)	98	43	42
						*Psolus* sp. (Sea cucumber)	7	Zoanthidae (Zoanthid)	95	43	41
						*Ophiopleura* sp. (Brittle star)	79	*Scalpellum* sp. (Barnacle)	100	36	36
Hard substrate	9	125 (22–60)	8.11 (11.30)	289 (179)	**9/0**	Porifera (Sponges)	67	Porifera (Sponges)	93	67	62
						*Strongylocentrotus* sp. (Urchin)	100	*Ophiacantha* sp. (Ophiuroid)	49	100	49
						*Actinauge* sp. (Anemone)	33	*Ophiopus* sp. (Ophiuroid)	69	44	31
						*Gorgonocephalus* spp. (Basket star)	56	*Strongylocentrotus* sp. (Urchin)	29	100	29
						*Ophiacantha* sp. (Brittle star)	100	*Eurycyde* sp. (Pycnogonid), *Glycera* sp. (Polychaete: Glyceridae), *Halice* sp. (Amphipod)	100	22	22
Shelf break	13	140 (10–63)	1.35 (2.02)	**180 (92)**	3/10	*Ophiocten* sp. (Brittle star)	85	*Yoldiella* spp. (Bivalve)	78	46	36
						*Urasterias* sp. (Sea star)	15	*Laonice* sp. (Polychaete: Spionidae)	85	31	26
						*Hyas* sp. (Crab)	8				
						*Nuculana* spp. (Bivalve)	46				
						*Saduria* spp. (Isopod)	15				
Local hotspots	13	206 (15–86)	**21.54 (21.82)**	301 (233)	3/10	*Gorgonocephalus* spp. (Basket star)	38	Nephtheidae (Soft corals)	61	92	56
						Dendrochirotida (Sea cucumber)	38	*Phascolion* sp. (Sipuncula: Phascolionidae)	91	54	49
						*Balanus* sp. (Barnacle)	23	*Buccinum* spp. (Gastropod)	79	62	49
						*Strongylocentrotus* sp. (Urchin)	23	*Nymphon* spp. (Pycnogonid)	59	77	45
						*Ophiopleura* sp. (Brittle star)	38	*Eualus* spp. (Decapod)	73	54	39

For each community cluster, the five dominant taxa in terms of biomass and the five most significant indicator taxa (*p*<0.05) ranked according to their indicator value index (IndVal) are shown.

SD: standard deviation. ‘IndVal’ index is a measure of association between a taxon and a cluster of stations and is calculated as the product of specificity (mean biomass of a given taxon within a cluster compared to the other clusters) and fidelity (taxon occurrence at stations belonging to a cluster).

### 3.3. Environmental drivers of community clusters

**Figure 7:**
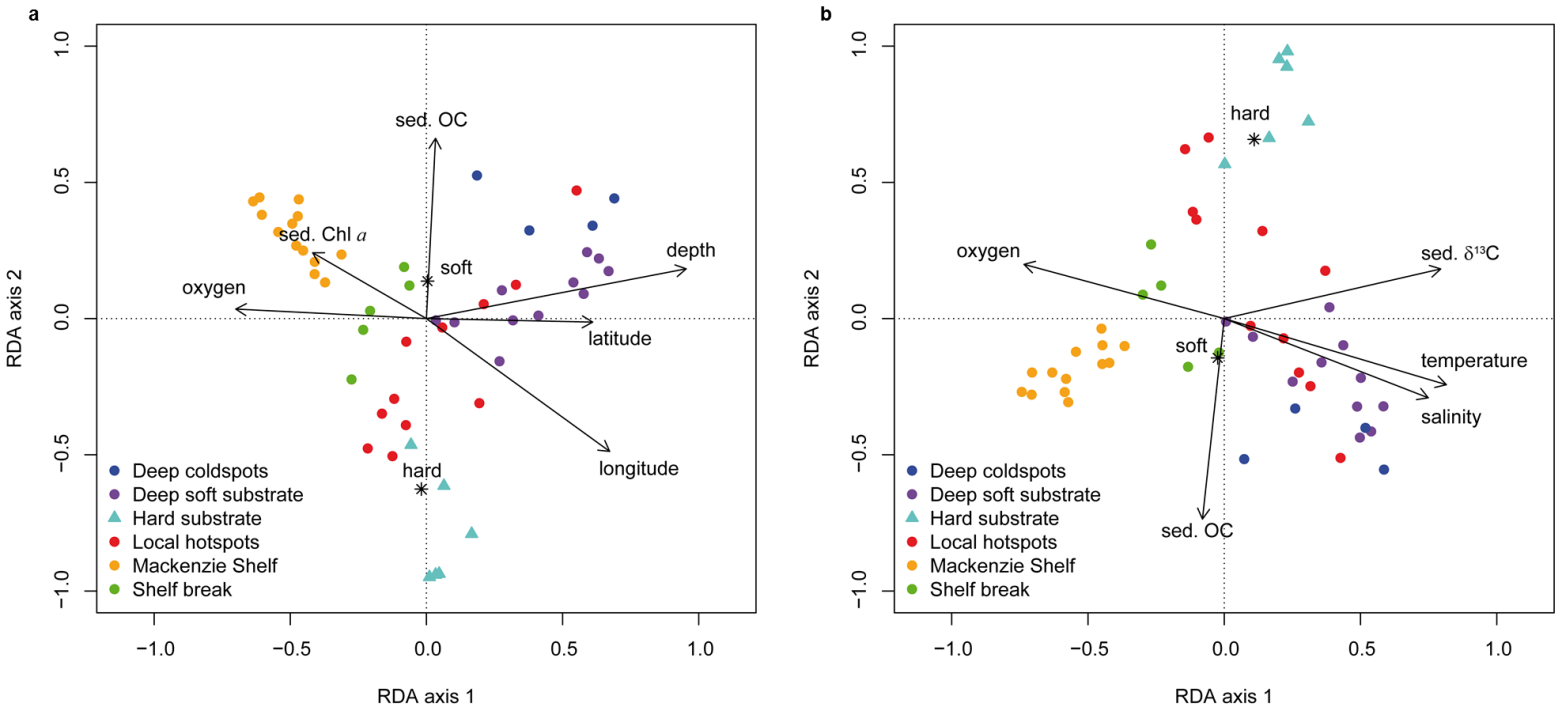
Redundancy analysis (RDA) ordination plots of megabenthic biomass-based taxonomic composition against forward selected environmental variables (black arrows and centroids) on 50 stations from 2008 to 2011. (**a**) Including indirect/spatial variables; the first two RDA axes explained 61% of the variance. (**b**) Without spatial variables; the first two RDA axes explained 64% of the variance. The categorical variable ‘substrate type’ is illustrated using centroids (*) for each category (hard and soft) and colors represent the six benthic communities defined in this study.

Community clusters were significantly influenced by a set of environmental variables that explained between 19% and 22% (R^2^_adjusted_) of the variation in the RDA analysis. These are low but typical variance levels explained for biological systems [61], as the high complexity of these systems rarely makes it conceivable to encompass all the variables that balance the responses of organisms or communities [62]. Among the fifteen explanatory variables (including indirect/spatial variables) employed in the forward selection of the RDA model on 50 stations, seven variables were retained (Table 4, Figure 7a). The final model significantly explained 22% of the taxonomic composition variation (R^2^ = 0.33, R^2^_adj_ = 0.22). Depth, longitude, latitude, sediment Chl *a* and bottom oxygen were strongly correlated with the first RDA axis, while substrate type and sediment organic carbon were highly correlated with the second RDA axis (Table 4, Figure 7a). Among the twelve explanatory variables included in the forward selection of the RDA model excluding indirect/spatial variables, six were retained (Table 4, Figure 7b). The final model significantly explained 19% of the mega-epibenthic taxonomic composition variation (R^2^ = 0.29, R^2^_adj_ = 0.19). Bottom salinity, oxygen, temperature and sediment δ^13^C were strongly correlated with the first RDA axis, while substrate type and sediment organic carbon were highly correlated with the second RDA axis (Table 4, Figure 7b). The first RDA axes of both models reflected mostly the distribution of community clusters along two large-scale environmental gradients (100–1000 km): (1) a vertical gradient created by depth, bottom oceanographic variables and sediment Chl *a* variables, and (2) a geographical gradient generated by longitude, latitude and sediment δ^13^C variables. The second RDA axes of the models reflected the distribution of community clusters along a meso-scale environmental gradient (10–100 km) of the sedimentary environment characterized by the variables substrate type and sediment organic carbon. The six community clusters obtained from the unconstrained Ward clustering analysis (Figure 4) were well segregated within the RDA models, except the ‘local hotspots’ community cluster with stations scattered along the second RDA axes. The sediment organic carbon content recorded within this community was highly variable, from high values underneath NOW polynya and in Barrow Strait to low values in Victoria Strait and off Cape Bathurst (Table S2 in File S1).

**Table 4:** Redundancy analysis (RDA) results on relationships between megabenthic biomass-based taxonomic composition and environmental variables for a subset of 50 stations sampled from 2008 to 2011.

With spatial variables			Without spatial variables		
	RDA axis 1	RDA axis 2		RDA axis 1	RDA axis 2
Eigenvalue	0.09	0.07	Eigenvalue	0.09	0.06
Variance explained	0.35	0.26	Variance explained	0.37	0.27
					
Correlations with environmental variables			Correlations with environmental variables		
Depth	0.95	0.18	Bottom temperature	0.82	−0.24
Substrate (hard)	−0.02	−0.63	Substrate (hard)	0.11	0.66
Substrate (soft)	0.00	0.14	Substrate (soft)	−0.02	−0.14
Longitude	0.67	−0.49	Sediment δ^13^C	0.79	0.18
Latitude	0.61	−0.01	Bottom salinity	0.75	−0.29
Sediment Chl *a*	−0.42	0.24	Sediment organic carbon	−0.08	−0.74
Sediment organic carbon	0.03	0.66	Bottom oxygen	−0.73	0.20
Bottom oxygen	−0.70	0.03			

Two RDA analyses were performed, with and without spatial variables, and both were significant (*p*<0.001; 9999 permutations). Results from the first two RDA axes are shown and environmental variables are listed in order following forward selection (9999 permutations).

## Discussion

This study represents the first continental-scale assessment of the taxonomic composition of megabenthic communities and the relationships of various environmental factors acting at different spatial and temporal scales to their community structure. As we hypothesized, benthic univariate community characteristics had a tendency to decrease with depth and increase with food supply proxies, but few correlations were significant. Distribution patterns of community clusters were significantly associated with large- and meso-scale environmental factors, but again explanatory power of the models was moderate. We discuss how local- to meso-scale environmental conditions in specific locations of the Canadian Arctic disrupt the hypothetical large-scale trends we expected to observe with depth and food supply proxies. We conclude that broad generalizations based on these community-environment relationships over the large geographical extent of the Canadian Arctic are not straightforward unless predictions take into account the influence of local- to meso-scale environmental conditions at some locations.

### 4.1. Environmental drivers of community structure

**Figure 8:**
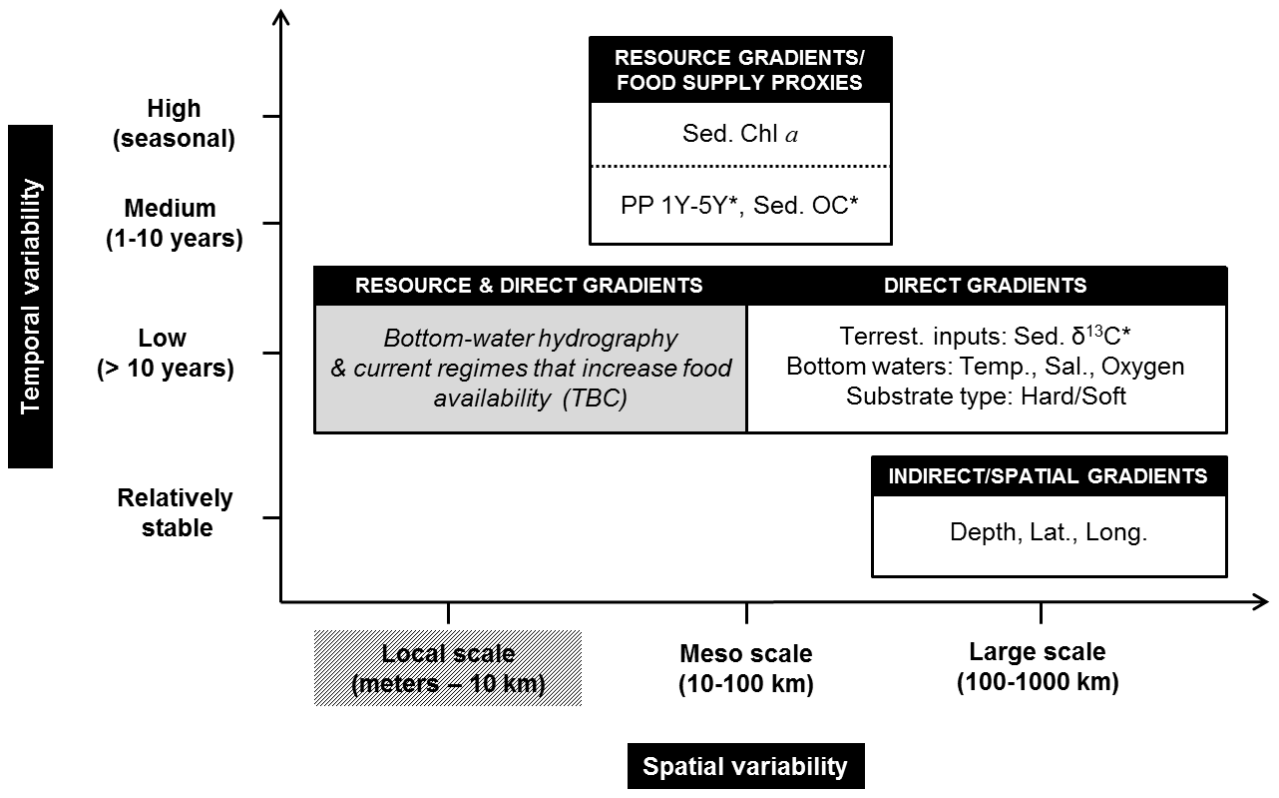
Conceptual figure displaying the overall results of environmental drivers of megabenthic communities in this study in relation to their spatial and temporal scales of variability; potential missing important drivers (gray box) would have to be confirmed (TBC). Environmental factors available for the present study were divided into three categories: resource, direct and indirect/spatial gradients (following [14]). Sampling design of the present study prevented conclusion at local scale (dashed). * denotes environmental variables that were either significantly correlated with univariate community characteristics or to community cluster distribution.

We propose a conceptual model illustrating spatial and temporal scales of variability of the significantly retained environmental drivers of megabenthic community characteristics and cluster distribution (Figure 8). All direct and indirect/spatial gradient variables considered were significantly related to community characteristics and/or cluster distribution. Among several food supply proxies tested, four were significantly retained, i.e., sediment Chl *a*, sediment organic carbon, PP 1Y and PP 5Y. We refer to this conceptual model in the discussion below.

#### 4.1.1. Large-scale environmental gradients

The decrease of benthic biomass and density with depth in the World's oceans in general (e.g., [10], [63], [64]), and in Arctic systems specifically (e.g., [16], [20], [24], [65], [66]), has been commonly acknowledged to be a reflection of the vertical decline of organic material flux reaching the seafloor [67], [68]. This link to declining food deposition can be seen in the present study by the negative correlation between sediment Chl *a* and depth. The positive correlation we found between sediment Chl *a* and the absolute amount as well as the relative proportion of large phytoplankton cells (≥ 5 µm) support the fact that large, rapidly-sinking cells contribute most to the carbon flux reaching the seafloor [49]. The parallel significant declines of sediment Chl *a* and benthic biomass with depth support that deep communities sampled in this study were likely constrained by the supply of fresh organic matter. The strength of the correlations was, however, only moderate (correlation coefficients<0.5), meaning that the assumption of decreasing food supply, benthic biomass and density with depth is not necessarily straightforward for the entire Canadian Arctic. The weak decreasing trend of benthic biomass and density with depth over the study area is mostly driven by several biomass-rich and density-rich deep stations (>200 m) located in the Lancaster Sound-Bylot Island (LS-BI) and NOW polynyas. The strong pelagic-benthic coupling in deep areas of the Eastern Canadian Arctic relative to weak pelagic-benthic coupling in deep areas of the Western Canadian Arctic has also been observed by the spatial variability in the magnitude of benthic boundary fluxes [37].

In addition to biomass and density, biodiversity metrics (i.e., S_density_ and H′) also varied or had a tendency to vary with depth in our study. Depth was strongly linked to physical properties of water masses (salinity, temperature, oxygen) and it is possible that the vertical Pacific/Atlantic water mass gradient may explain in part, beside declining food supply with depth, the depth-related gradients in benthic biodiversity metrics and taxonomic composition. Possible factors, alone or in combination, associated with distinct water masses and that may contribute to benthic diversity patterns include: Influence of physical discontinuities in the water column on distributional patterns of invertebrate larvae [69]; physiological tolerances of benthic organisms to hydrographic conditions [10]; and the geological history such as post-glaciation events that promoted the colonization of American Arctic shelves by species from the Pacific and Atlantic oceans [70]. We cannot tease apart the relative influence of these factors or the influence of depth versus water mass, but our results convincingly demonstrated that the two ‘deep’ community clusters were taxonomically more similar than with shallower community clusters. Bottom oceanographic variables also largely structure benthic communities in other Arctic regions, such as the East Greenland shelf and slope [24], [71], the northeastern Chukchi Sea [72], [73], and in the Barents Sea [23], [74], [75]. Decline in biodiversity with depth also may in part be explained by decreasing availability of fresh food with depth, similar to biomass and density. For example, as food supply decreases, richness may decrease because fewer species can maintain viable populations [10]. We did not, however, find significant correlations either between primary productivity proxies in surface waters and benthic biodiversity metrics or between sediment food supply proxies and benthic biodiversity metrics, making this link likely less important than the control of physical properties of bottom waters.

The sediment δ^13^C gradient replaced the spatial variable longitude when the latter was excluded from the RDA model, revealing the influence of terrestrial organic matter inputs on taxonomic composition. Sediment δ^13^C exposed a large-scale geographical gradient in taxonomic composition with the majority of communities under terrestrial influence located in the western Canadian Arctic near the Mackenzie River drainage (‘Mackenzie Shelf’ cluster) or in the coastal/shelf region of the Amundsen Gulf (‘shelf break’ cluster). The decrease of the contribution of terrigenous organic matter towards the eastern Canadian Arctic has been also documented by various sedimentary biomarkers [76]. The refractory proportion of allochthonous organic carbon delivered by Arctic rivers is high [77] and terrestrially-derived carbon is typically of low food quality for marine consumers [7]. The lack of correlations between sediment δ^13^C and total benthic biomass and density, and the high benthic biomass observed on the Mackenzie Shelf, however, do not support this effect of low food quality. Therefore, in the present context bulk sediment δ^13^C did not indicate on the whole what benthic organisms were consuming and was correctly defined as not relevant in the resource gradients. Possibly, effects of terrestrial influence differ by species or feeding type, thus influencing taxonomic composition but not bulk biomass or density.

#### 4.1.2. Meso-scale environmental gradients

Highly biologically productive areas, such as polynyas, are generally thought to favor benthic systems [78], [79]. Across the Canadian Arctic, the presence of polynyas was not reflected on the benthos by a change in community structure in this study, with the exception of the LS-BI and NOW polynyas. Variation in primary productivity (in magnitude and composition) and in zooplankton grazing pressure among the Canadian Arctic polynyas likely result in variable carbon supply to the benthos, precluding generalizations. In this study we considered the influence of polynyas only for those stations located directly underneath, but these meso-scale oceanographic features also may have a significant influence on benthic community structure in surrounding areas because of the advective transport of organic material by currents [80]. We were not able to assess the marginal effects of polynyas in this study (e.g., by means of particle interceptor traps) and the great water depths (>200 m) underneath the polynyas may have enhanced the advection of POC. This would be supported by the higher sediment Chl *a* concentrations found in absence than presence of polynyas in this study (results not shown), but this pattern may again be confounded by the shallower depth of non-polynya stations.

Bulk benthic biomass and density were significantly correlated with 1-year and 5-year integrated PP estimates in surface waters, but not with *in situ* measurements of euphotic phytoplankton biomass, possibly due to the mismatch between a short-term estimate of the primary productivity and its export and the integrated, long-term benthic community responses. Among the few benthic studies in the Arctic using integrated PP estimates as a food supply proxy, a positive correlation has been established for macrofaunal density [74], [75] in the Barents Sea where seasonal food freshness indicators on the seafloor, such as sediment Chl *a*, were also positively correlated with macrobenthic biomass and density [23], [74], [75], [81]. PP estimates did not, however, significantly explain the taxonomic composition variation of megabenthic communities in the present study, contrary to what was observed for macrobenthic communities in the Barents Sea [75]. While sediment organic carbon and sediment phaeopigments have often been significantly correlated with macrobenthic biomass, density, species richness and Shannon-Wiener's diversity [23], [74], [82], [83], they have typically not been related to megafaunal community characteristics and taxonomic composition [2], [71], [84], and also not in this study. Sediment organic carbon contains large fractions of refractory material [47] and, along with sediment phaeopigments, reflects mid- to long-term organic matter inputs to the seafloor, thus likely representing unattractive organic matter sources. Settlement of fresh material, as seen by the positive influence of sediment Chl *a* on bulk biomass, may be critical for Arctic megabenthic communities, possibly because of the substantial metabolic energy required on an individual basis by large organisms [64]. One missing but highly relevant organic matter source that we could not approximate in the present study is the organic matter pool derived from sea-ice algal communities. Arctic benthic communities may rely heavily on this food source [85] and it thus may explain the limited correlation of the primary productivity estimates in open waters in the present study with benthic community structure. We did not have any direct measures of sea ice algae and the complexity of environmental constraints on sea-ice algal biomass (e.g., ice thickness, snow cover, nutrient concentrations; [86]) did not allow us to estimate export of ice algal biomass from proxies. However, the presence of IP_25_, an ice algal biomarker, in the surface sediment of seven stations occupied across the study area documents that ice algal export occurred in the study region [87].

Hard substrates in this study had lower S_density_ and H′ than soft substrates, although they generally provide higher habitat complexity than soft substrates and thus tend to house a larger number of species [88]. This negative relationship of hard substrate bottom on biodiversity may be due to the low number of hard substrate stations sampled in this study and also because organisms were heavily damaged by rocks during trawling, thereby making taxonomic determination arduous. Substrate type, however, significantly explained the variation of taxonomic composition, as also demonstrated in other Arctic studies [2], [24]. We propose that, in the context of the present study, substrate type along with sediment organic carbon were mostly indicative of the meso-scale sedimentary environment variability on the second RDA axes with higher deposition of organic carbon in soft substrate than in hard substrate bottoms (see the opposite direction of hard substrate and sediment organic carbon, Figure 7). Sediment organic carbon and substrate variability can be indirect indicators of current transport and sedimentation zones, thus influencing the type of benthic fauna occupying a region [83]. These two environmental factors did not, however, explain well the local- to meso-scale conditions of the sedimentary environment in the ‘local hotspots’ community type, with stations of this community scattered along the second RDA axes. It is likely that other, unmeasured environmental factors, such as water currents and bottom topography, could explain the distribution of the ‘local hotspots’ community (Figure 8, also see below).

### 4.2. Distribution patterns of community clusters

The majority of the community clusters were observed throughout the extent of the study area, except ‘Mackenzie Shelf’ and ‘deep coldspots’ community clusters, which were restricted to the western Canadian Arctic. A similar geographic segregation was previously observed for the zoogeography of marine bivalves of the Canadian Arctic waters [89], confirming an important zoogeographic boundary in the western sector of the Canadian Archipelago between the faunas of the Atlantic and the Pacific sectors. The presence of an independent evolutionary trend in this region caused by Pleistocene isolation, along with the narrow, abrupt shelf of the western Archipelago and the zone of brackish waters at the mouth of the Mackenzie River are all potential barriers to faunistic interchange [89]. This likely shaped, albeit to an unknown degree, the west-to-east variation in taxonomic composition observed in this study.

#### 4.2.1. ‘Mackenzie Shelf’ community

Community structure in this cluster was most dissimilar to all other clusters, with indicator taxa that were almost exclusively restricted to it. This high faunal distinctiveness is conceivably related to the high terrestrial carbon and freshwater influxes from the Mackenzie River and to the shallow depth range of this community cluster. Among the indicator taxa identified in this community type, the isopod *Saduria* spp., is euryhaline [90] and the two indicator bivalve taxa, *Ciliatocardium* sp. and *Macoma* spp., are specific to shallow waters of Arctic shelf areas [89]. The high specificity and fidelity of these indicator taxa to this community reflected the strong influence by the Mackenzie River. The particular environmental forcing exerted on Arctic benthic community composition by large river inflow geographically is known to structure macrofaunal community distribution patterns [39], [91]–[94], and, as we demonstrated here, also megafaunal distribution patterns. The distinct oceanographic, physical and biological properties of large rivers draining onto Arctic shelves create quasi-independent systems, as observed for the Pechora Sea in the southeast Barents Sea [92], [95].

#### 4.2.2. ‘Shelf break’ community

This community was found mostly in the Amundsen Gulf region, but also in the Archipelago and in Baffin Bay. The lower benthic biomass and density recorded in this community compared to other community types found at similar depth ranges (i.e., ‘local hotspots’ and ’hard substrate’ clusters) may indicate weaker pelagic-benthic coupling and/or lower food quality as this community was located generally in coastal areas of narrow shelves where terrestrial organic matter inputs were high (as indicated by low sediment δ^13^C). However, in a parallel study carried out at several stations of this community in Amundsen Gulf region, sediment Chl *a* concentrations and benthic carbon remineralization were above the regional average, indicating relatively tight pelagic-benthic coupling compared to the central Amundsen Gulf region [40]. The ‘shelf break’ community was located at a transitional zone between the shelf and the slope but also between the Pacific and the Atlantic water masses, both transitions being around 200 m [26]. The environmental conditions around the shelf break could have generated specific and strong habitat heterogeneity. Physical disturbances may be high for the benthic habitat in coastal areas of the Amundsen Gulf where the narrow shelf is subjected to intense erosion and is influenced to the west by the Mackenzie River sediment load discharge [28]. The detrivorous feeding behavior of the two indicator taxa of this community, the bivalve *Yoldiella* spp. and the spionid polychaete *Laonice* sp. [96], supports the notion of high sediment deposition but additional studies are needed to assess the relative influence of either physical discontinuities in the water column or seafloor erosion in shaping this community type.

#### 4.2.3. ‘Deep coldspots’ community

Many stations of this community were located under the CB polynya as well as under a phytoplankton-based eutrophic hotspot defined by Ardyna et al. [27] in the central Amundsen Gulf, revealing the absence of a specific influence of this particular polynya on megafaunal communities. Similarly, this polynya did not influence taxonomic composition of macrofaunal communities [39] and low rates of carbon remineralization and benthic boundary fluxes have been measured in this polynya [22], [37], [40]. It has been proposed recently for the central Amundsen Gulf that the pelagic food web may intercept a major part of the POC before it reaches the seafloor [22], [40], [97], thus dampening the pelagic-benthic coupling in this area. In support of this, high concentrations of degraded pigments were present in surface sediments of the Amundsen Gulf than on the adjacent Mackenzie Shelf [45].

#### 4.2.4. ‘Deep soft substrate’ community

Contrary to the ‘deep coldspots’ community, the ‘deep soft substrate’ community was mostly located in the Eastern Canadian Arctic under the productive NOW and LS-BI polynyas [27], [35], where strong pelagic-benthic coupling has been reported [36], [37], [40], [65]. While the CB polynya in general does not seem to favor strong pelagic-benthic coupling (see above), the assemblage of several ‘deep soft substrate’ stations at the western edge of the polynya seems to indicate local- to meso-scale patterns of strong pelagic-benthic coupling at the western polynya margin. Wind-driven upwelling occurs near the CB polynya [98], which promotes high nutrient replenishment for primary production [99] and strong vertical POC flux [97], [100]. This high productivity and tight pelagic-benthic coupling is also reflected in the high ampeliscid amphipod biomass in that region [101]. The large biomass of anemones found at several stations of this community probably reflects regionally specific bottom-water hydrography and/or current regime replenishing their food supply. For instance, strong currents at the eastern deep entrance of Lancaster Sound [102] have been acknowledged to maintain high benthic communities of filter feeders [65].

#### 4.2.5. ‘Hard substrate’ community

This community cluster was mostly present in the Eastern Canadian Arctic, but this might be biased by the sampling distribution, as stations with hard substrata were usually avoided. The most significant indicator taxa were suspension feeders, indicative of a strong current regime [78]. Not all hard substrate stations were grouped in this community cluster, however, suggesting that a more complete substrate classification would improve our understanding of the influence of substrate variability on taxonomic composition. In landscape-scale studies, terrain variables such as slope and roughness may explain a significant proportion of the community structure variation [88], [103]. Unfortunately, habitat descriptions based on videos/images and on acoustic techniques (e.g., multibeam data), including near-bottom flow conditions, are scarce in the Canadian Arctic, and future studies are needed to gain more habitat complexity information.

#### 4.2.6. ‘Local hotspots’ community

The combination of significant environmental drivers retained in the RDA models did not explain well the distribution of the ‘local hotspots’ community cluster, scattered along the second RDA axes showing a meso-scale gradient (10–100 km) influence of the sedimentary environment. We propose that high biomass and taxonomic similarity among the stations within this cluster may originate from unmeasured local- to meso-scale physical and biological conditions that promoted tight pelagic-benthic coupling and/or lateral advection of suspended particles (Figure 8). In the northeastern Chukchi Sea, it has been recently proposed that macro- and megafaunal community structure are influenced by local-scale topographically-driven water circulation that causes variation in organic carbon deposition [73], [104], [105]. Our data along with previous findings provide supporting evidence for an association between specific bottom-hydrography and/or current regime and the biological characteristics of this community. For instance, soft corals (Nephtheidae) were quasi-omnipresent in this community cluster (in 92% of stations) and thrive in regions with suspended food particles delivered by strong currents [106]. In the Eastern Canadian Arctic, for example, the NOW polynya has elevated primary production [8], [107], [108], high flux of organic matter [48], [109], [110], and high carbon remineralization and benthic boundary fluxes [36], [37] that coincide with a high number of megabenthic ‘local hotspots’ stations in that region. Moreover, many ‘local hotspots’ stations were found in the western-central section of the NOW polynya where a strong southward flow of deep, cold water from the Arctic Ocean prevails, while ‘deep soft substrate’ communities in the deeper eastern section of the polynya were positioned under the weak northward flow of the warmer West Greenland Current [111], re-emphasizing the influence of hydrographic regime. One ‘local hotspot’ community was located in the east off Cape Bathurst, where local upwelling [98] is likely to have caused this one station to have the highest biomass recorded in this study and be the only ‘local hotspot’ community station in the western sector of the study area. Other ‘local hotspots’ stations distributed across the study region were not located in areas of high annual pelagic primary production [35] and had low sediment organic carbon values. However, high tidal currents may favor the transport of large amounts of organic matter to the seafloor or resuspension of material, such as in Victoria Strait (station 312, Figure 5) and Barrow Strait (station 304, Figure 5) [26], [112]. This is in agreement with the high biomass of crinoids, passive suspension feeders [106], found in Victoria Strait and the high carbon remineralization rates and benthic boundary fluxes at the ‘local hotspot’ in Barrow Strait [36], [37]. To our knowledge, no information on the current regime exists for the ‘local hotspot’ station of Gibbs fjord (station GF2, Figure 5). However, this station had a high density of holothurians (*Elpidia* sp.), which have been suggested to be indicative of fresh phytodetritus pulses [2], [19], [113], [114]. Our limited understanding of the environmental controls on the ‘local hotspots’ community defined in this study emphasizes the need to better describe local- to meso-scale bottom-water hydrography and current regimes that could favor high advection of organic material (Figure 8). This is especially true in areas of the central Archipelago where primary productivity is low [27], [35], giving rise to the sometimes wrong assumption that food supply for benthic communities would also be low.

## Summary and Implications

The central role of food supply in shaping various benthic community attributes is a central subject of current research in the Arctic as it may be most affected by future climate changes [9]. This study revealed, however, that broad generalization of the pelagic-benthic coupling strength may not always be straightforward, as surface production was not always a good predictor of community structure in our study region across the Canadian Arctic. Depth also was not strongly related to benthic biomass and density over the extent of the Canadian Arctic, likely because meso-scale processes enhanced the food supply for deep benthic communities at some locations, particularly underneath highly productive polynyas. None of the food supply proxies that we included in this study was strongly correlated with benthic community characteristics and taxonomic composition, because none reflected the strength of the pelagic-benthic coupling over the entire geographical extent of the Canadian Arctic. For instance, we argue that low-biomass benthic communities received low food supply in eutrophic areas due to strong pelagic interception of POC fluxes (e.g., CB polynya in the Western Canadian Arctic), while biomass-rich benthic communities were found in oligotrophic areas because of specific local- to meso-scale bottom-water hydrography and/or current regimes likely favoring high advection of organic material (e.g., central Canadian Arctic Archipelago). Local- to meso-scale investigations of water circulation that could influence lateral advection of organic material will improve our ability to understand the environmental controls on Canadian Arctic benthic communities to better understand present patterns, and eventually predict future responses of the Canadian Arctic benthic communities in a changing environment. Finally, the various spatial scales of the environmental gradients influencing benthic communities may benefit the processes of delineating and characterizing Ecologically and Biologically Significant Areas (EBSAs) [36] within large biogeographic regions of the Canadian Arctic that are primarily based on oceanographic and bathymetric similarities [115].

## Supporting Information

File S1 **Supporting figures and tables. Figure S1**, Principal Component Analysis (PCA) plot showing the multivariate variation among 50 stations in terms of environmental variables. **Figure S2**, Station-based variation in mean relative (%) biomass composition and taxonomic composition for the main phyla sampled across all community clusters. **Table S1**, Megabenthic community characteristics for the 78 stations sampled from 2007 to 2011 across the Canadian Arctic. **Table S2**, Environmental variables for the 78 stations sampled from 2007 to 2011 across the Canadian Arctic. **Table S3**, Faunal inventory of all megabenthic taxa identified at the lowest possible taxonomic level across the Canadian Arctic.
(PDF)
