# Supplementary material for: Environmental Drivers of the Canadian Arctic Megabenthic Communities
Source: PLoS One. 2014 Jul 14;9(7):e100900. doi: 10.1371/journal.pone.0100900 (PMC4096404; doi:10.1371/journal.pone.0100900)
Supplement: File S1 — Supporting figures and tables. Figure S1, Principal Component Analysis (PCA) plot showing the multivariate variation among 50 stations in terms of environmental variables. Figure S2, Station-based variation in mean relative (%) biomass composition and taxonomic composition for the main phyla sampled across all community clusters. Table S1, Megabenthic community characteristics for the 78 stations sampled from 2007 to 2011 across the Canadian Arctic. Table S2, Environmental variables for the 78 stations sampled from 2007 to 2011 across the Canadian Arctic. Table S3, Faunal inventory of all megabenthic taxa identified at the lowest possible taxonomic level across the Canadian Arctic. (PDF) [file pone.0100900.s001.pdf]

The following supplement accompanies the article

Environmental drivers of the Canadian Arctic megabenthic communities

Virginie Roy[1*], Katrin Iken[2] and Philippe Archambault[1]

[1] Institut des sciences de la mer de Rimouski, Université du Québec à Rimouski, Rimouski, Québec, Canada

[2] School of Fisheries and Ocean Sciences, University of Alaska Fairbanks, Fairbanks, Alaska, USA

*Corresponding author email: virginie.m.roy@gmail.com

Table S1. Megabenthic community characteristics for the 78 stations sampled from 2007 to 2011 across the Canadian Arctic. Stations are ordered by year and month. na: data not available.

			Benthic community characteristic							
**Station code**	**Year**	**Month**	**Community cluster**	**Biomass**	**Density**	**Taxononomic richness density**	**Taxononomic richness**	**Shannon-Wiener index**	**Pielou's evenness**	**Average taxonomic distinctness**
**ArcticNet label_year**				**(g wet mass m^{-2})**	**(ind. m^{-2})**	**$S_{density}$ (no. of taxa 1000 m^{-2})**	**(no. of taxa station^{-1})**	**H'**	**J'**	**Δ***
1100_07	2007	Oct	Shelf break	0.18	0.16	27.36	19	1.80	0.61	93.3
1214_07	2007	Oct	Deep soft substrate	1.64	3.71	54.72	19	2.06	0.70	95.2
1216_07	2007	Oct	Deep soft substrate	30.56	65.89	31.68	11	1.69	0.71	93.6
1800_07	2007	Oct	Deep coldspots	0.83	0.49	27.36	19	0.93	0.32	99.4
302_07	2007	Oct	Hard substrate	0.89	0.41	47.52	33	2.13	0.61	91.2
405_07	2007	Oct	Deep coldspots	0.03	0.17	15.84	11	1.99	0.83	91.8
408_07	2007	Oct	Deep soft substrate	3.92	2.21	48.96	17	1.18	0.42	87.2
420_07	2007	Oct	Local hotspots	77.09	119.11	77.75	27	1.44	0.44	95.6
434_07	2007	Oct	Mackenzie Shelf	1.75	1.60	61.91	43	2.62	0.70	87.6
437_07	2007	Oct	Deep coldspots	0.73	0.82	71.99	25	2.05	0.64	88.4
FB3_08	2008	Jun	Shelf break	0.21	0.08	25.92	18	1.58	0.55	98.8
F7_08	2008	Jun	Shelf break	0.43	0.32	46.40	29	2.31	0.69	93.2
1200_08	2008	Jun	Shelf break	0.51	1.32	71.99	25	2.03	0.63	97.6
1208_08	2008	Jun	Deep coldspots	1.10	0.34	49.60	31	0.55	0.16	97.4
434_08	2008	Jun	Mackenzie Shelf	0.35	0.25	71.99	25	2.74	0.85	92.5
1110_08	2008	Jul	Shelf break	3.17	2.83	69.11	24	1.63	0.51	91.2
2010_08	2008	Jul	Deep coldspots	0.16	0.30	22.80	19	1.91	0.65	90.5
D34_08	2008	Jul	Shelf break	0.12	0.17	23.04	16	1.50	0.54	92.7
410_08	2008	Jul	Deep coldspots	2.01	0.78	33.12	23	1.26	0.40	71.8
416_08	2008	Jul	Shelf break	0.59	0.65	53.48	26	1.73	0.53	93.1
435-10A_08	2008	Aug	Deep coldspots	1.29	0.60	54.00	21	1.24	0.41	84.3
140_08	2008	Sep	Shelf break	0.94	0.31	48.96	17	1.31	0.46	89.4
205_08	2008	Sep	Local hotspots	3.42	2.40	54.00	15	0.48	0.18	95.3
233_08	2008	Sep	Deep soft substrate	4.13	0.81	89.99	25	1.62	0.50	85.8
304_08	2008	Sep	Shelf break	2.41	1.52	176.38	49	2.28	0.59	94.7
109_09	2009	Oct	Local hotspots	6.76	3.28	129.59	36	1.51	0.42	92.5
115_09	2009	Oct	Deep soft substrate	0.21	0.44	55.05	13	2.08	0.81	95.1
437_09	2009	Oct	Shelf break	0.33	0.51	33.23	10	1.08	0.47	95.8
450_09	2009	Oct	Shelf break	0.84	2.29	193.35	47	1.59	0.41	98.4
301_10	2010	Aug	Deep soft substrate	5.22	6.33	184.30	64	2.10	0.51	94.2
305_10	2010	Aug	Hard substrate	4.78	1.64	110.69	41	1.24	0.33	97.6
310F_10	2010	Aug	Hard substrate	1.99	4.28	138.23	48	2.26	0.58	93.0
312_10	2010	Aug	Local hotspots	2.64	2.41	226.78	84	2.59	0.58	94.8
105_10	2010	Oct	Local hotspots	23.38	5.89	230.38	80	1.45	0.33	90.6
108_10	2010	Oct	Local hotspots	27.35	4.46	227.50	79	1.56	0.36	90.7
111_10	2010	Oct	Local hotspots	7.76	1.10	43.20	30	2.03	0.60	79.8
115_10	2010	Oct	Hard substrate	4.48	1.10	172.78	60	1.92	0.47	90.5
304_10	2010	Oct	Local hotspots	13.41	2.01	115.19	40	1.00	0.27	88.1
323_10	2010	Oct	Deep soft substrate	10.04	7.26	88.93	35	1.69	0.48	96.7
405_10	2010	Oct	Deep coldspots	0.59	0.57	55.79	31	1.38	0.40	98.2
450_10	2010	Oct	Shelf break	7.34	14.97	151.19	63	2.38	0.57	89.6
175_11	2011	Jul	Hard substrate	2.66	0.76	64.79	30	1.73	0.51	88.3
150_11	2011	Aug	Hard substrate	14.69	1.33	119.99	30	1.30	0.38	96.8
160_11	2011	Aug	Deep soft substrate	0.72	2.36	69.11	32	2.28	0.66	91.7

310F_11	2011	Aug	Hard substrate	1.75	2.67	114.54	35	1.98	0.56	88.1
312_11	2011	Aug	Local hotspots	1.19	1.59	187.39	59	2.44	0.60	95.1
13_11	2011	Sep	Mackenzie Shelf	3.28	2.39	140.70	57	2.01	0.50	96.4
18_11	2011	Sep	Mackenzie Shelf	0.97	0.71	86.39	35	2.03	0.57	96.8
19_11	2011	Sep	Mackenzie Shelf	5.86	2.60	116.01	47	1.96	0.51	89.6
20_11	2011	Sep	Mackenzie Shelf	13.71	14.26	312.66	76	2.10	0.48	95.9
21_11	2011	Sep	Mackenzie Shelf	68.58	164.04	257.02	119	2.28	0.48	86.2
22_11	2011	Sep	Mackenzie Shelf	46.68	102.39	370.25	90	1.93	0.43	90.6
23_11	2011	Sep	Mackenzie Shelf	3.87	7.64	143.99	35	1.72	0.48	84.9
24_11	2011	Sep	Mackenzie Shelf	1.59	2.54	93.80	38	2.44	0.67	93.7
25_11	2011	Sep	Mackenzie Shelf	14.95	37.18	374.37	91	2.39	0.53	92.9
26_11	2011	Sep	Mackenzie Shelf	20.29	63.09	308.54	75	2.58	0.60	95.3
27_11	2011	Sep	Mackenzie Shelf	38.91	87.76	345.57	84	2.68	0.60	90.8
28_11	2011	Sep	Mackenzie Shelf	21.04	25.67	255.06	62	2.17	0.53	83.1
29_11	2011	Sep	Mackenzie Shelf	60.56	381.72	373.06	114	2.39	0.51	88.7
405_11	2011	Sep	Deep coldspots	0.06	0.77	149.75	52	2.68	0.68	96.1
420_11	2011	Sep	Local hotspots	52.52	12.56	311.97	65	1.40	0.34	98.4
437_11	2011	Sep	Hard substrate	5.54	4.20	63.35	22	1.99	0.64	94.8
438_11	2011	Sep	Mackenzie Shelf	13.47	59.44	302.37	63	2.27	0.55	94.5
460_11	2011	Sep	Mackenzie Shelf	2.03	6.17	123.83	43	2.29	0.61	91.4
466_11	2011	Sep	Deep coldspots	0.33	0.84	148.79	31	1.17	0.34	82.0
115_11	2011	Oct	Deep soft substrate	7.66	7.04	185.98	31	2.18	0.63	94.0
GF1_11	2011	Oct	Hard substrate	36.18	2.95	341.97	38	1.00	0.27	98.9
GF2_11	2011	Oct	Local hotspots	24.33	32.97	229.48	34	0.92	0.26	79.8
301_11	2011	Oct	Deep soft substrate	3.80	2.63	97.40	23	1.19	0.38	98.7
304_11	2011	Oct	Local hotspots	15.21	2.77	171.68	31	0.83	0.24	98.7
307_11	2011	Oct	Deep soft substrate	2.92	2.27	112.69	36	1.61	0.45	76.8
310M_11	2011	Oct	Deep soft substrate	0.33	0.97	57.59	16	1.72	0.62	97.0
312_3_11	2011	Oct	Local hotspots	24.94	19.77	247.66	86	3.21	0.72	94.6
314_11	2011	Oct	Shelf break	0.50	1.53	129.59	45	2.88	0.76	91.2
323_11	2011	Oct	Deep soft substrate	23.79	16.57	251.98	35	1.62	0.46	92.5
407_11	2011	Oct	Deep coldspots	1.33	5.66	143.99	30	1.84	0.54	94.5
408_11	2011	Oct	Deep soft substrate	2.50	1.40	104.18	41	2.15	0.58	88.5
472_11	2011	Oct	Deep coldspots	0.29	0.73	167.98	35	2.15	0.61	94.3

Table S2. Environmental variables for the 78 stations sampled from 2007 to 2011 across the Canadian Arctic. Stations are ordered by year and month. References (Ref) for published 2008-2009 sediment pigment data. Contacts for phytoplankton biomass data are: Michel Gosselin (michel_gosselin@uqar.ca ; Université du Québec à Rimouski, Rimouski, QC, Canada) and Jean-Éric Tremblay (jean-eric.tremblay@bio.ulaval.ca ; Université Laval, Québec, Québec, Canada). Contact for satellite-derived primary productivity (PP 1Y and 5Y) is: Simon Bélanger (simon_belanger@uqar.ca ; Université du Québec à Rimouski, Rimouski, Québec, Canada). na: data not available.

	**Indirect gradient**			**Direct gradient**					**Resource gradient**				
	**Spatial variable**			**Bottom oceanographic variable**					**Food supply proxy**				
**Station code**	**Lat °N**	**Long °W**	**Depth**	**Temperature**	**Salinity**	**Oxygen**	**Substrate**	**Sediment $\delta^{13}C$**	**Polynya**	**SedimentOC**	**Sediment phaeo**	**Sediment Chl *a***	**Ref**
**ArcticNet label_year**	**(DD)**	**(DD)**	**(m)**	**(°C)**		**(ml l^{-1})**	**(hard or soft)**	**(‰)**	**(yes or no)**	**(%)**	**(µg g^{-1})**	**(µg g^{-1})**	
1100_07	71.035	-123.261	276	0.24	34.72	5.44	soft	na	no	na	na	na	
1214_07	70.709	-127.322	222	0.23	34.69	5.78	soft	na	no	na	na	na	
1216_07	70.748	-127.913	72	-1.07	33.73	6.28	hard	na	no	na	na	na	
1800_07	72.187	-127.808	359	0.43	34.82	6.22	soft	na	no	na	na	na	
302_07	74.213	-86.650	488	0.70	34.37	5.37	hard	na	yes	na	na	na	
405_07	70.652	-123.030	619	0.35	34.77	4.93	hard	na	yes	na	na	na	
408_07	71.314	-127.641	194	0.22	34.67	5.83	soft	na	yes	na	na	na	
420_07	71.063	-128.398	46	-0.93	33.76	5.93	hard	na	yes	na	na	na	
434_07	70.173	-133.598	38	-1.78	32.41	8.14	soft	na	no	na	na	na	
437_07	71.803	-126.592	336	0.43	34.83	6.32	soft	na	yes	na	na	na	
FB3_08	69.974	-125.857	98	-1.33	33.25	7.09	soft	-26.6	no	1.44	9.14	1.36	[1,2]
F7_08	69.830	-123.627	84	-1.54	32.94	6.68	soft	na	no	na	na	na	
1200_08	71.543	-124.333	207	-0.27	34.38	4.82	hard	-24.2	no	1.73	11.97	0.86	[2,3]
1208_08	71.064	-126.170	401	0.32	34.83	6.17	soft	na	yes	na	na	na	
434_08	70.180	-133.551	45	-1.67	32.55	6.71	soft	-26.2	no	2.39	37.33	23.34	[2,4]
1110_08	70.321	-124.843	92	-1.46	32.93	6.15	hard	na	no	na	na	na	
2010_08	75.120	-120.421	424	0.39	34.80	4.59	hard	na	no	na	na	na	
D34_08	71.075	-121.817	184	-0.57	34.27	5.00	hard	na	no	0.43	10.83	0.74	[1,2]
410_08	71.708	-126.494	398	0.26	34.78	5.50	hard	na	yes	na	na	na	
416_08	71.311	-127.829	160	-1.48	33.38	5.74	soft	-25.4	yes	1.62	14.44	1.21	[3]
435-10A_08	71.082	-133.659	295	0.14	34.65	6.29	soft	na	no	na	na	na	
140_08	75.039	-64.474	272	1.83	34.20	5.58	soft	-23.1	no	0.69	5.27	0.28	[2]
205_08	77.221	-78.507	759	-0.23	34.25	6.17	soft	-22.5	no	1.32	15.42	1.14	[2]
233_08	76.740	-71.847	695	1.03	34.37	5.15	soft	-22.6	yes	1.84	27.06	3.62	[2]
304_08	74.277	-91.313	336	-0.25	33.83	5.33	soft	-21.8	no	2.41	46.01	14.14	[2,4]
109_09	76.284	-74.120	449	-0.17	34.39	6.22	soft	-22.8	yes	1.37	23.21	3.02	[4]
115_09	76.335	-71.258	562	-0.15	34.40	6.13	soft	-22.7	yes	1.69	20.15	1.06	[4]
437_09	71.795	-126.520	316	0.31	34.75	5.75	soft	-23.6	yes	0.98	3.46	0.16	
450_09	72.077	-119.812	96	-1.14	31.95	8.01	soft	na	no	na	na	na	
301_10	74.191	-83.994	671	0.98	34.45	4.10	soft	-21.5	yes	2.54	18.25	2.23	
305_10	74.312	-93.416	172	-1.32	32.85	6.27	hard	-21.3	no	0.81	8.88	1.97	
310F_10	71.296	-97.642	147	-1.19	32.98	5.77	hard	na	yes	na	na	na	
312_10	69.168	-100.678	55	-1.35	29.79	8.70	soft	-21.2	no	0.42	7.24	2.50	
105_10	76.286	-75.847	363	-0.16	34.30	6.30	hard	-21.6	yes	0.94	7.73	1.66	
108_10	76.229	-74.922	434	-0.14	34.34	6.28	soft	-21.6	yes	1.60	17.40	3.26	
111_10	76.284	-73.300	570	-0.13	34.35	6.31	soft	-21.6	yes	2.31	21.73	3.89	
115_10	76.360	-71.265	654	0.32	34.39	5.86	hard	-21.6	yes	1.86	14.12	1.55	
304_10	74.221	-91.552	305	-0.05	33.96	5.60	soft	-21.0	no	1.77	13.75	2.57	

323_10	74.203	-79.764	783	0.79	34.47	4.65	soft	-21.6	yes	2.20	11.13	1.02	
405_10	70.630	-123.000	616	0.35	34.77	5.88	soft	-22.8	yes	1.38	4.48	0.30	
450_10	72.050	-119.767	102	-1.29	32.50	6.73	soft	-22.4	no	1.18	14.10	4.11	
175_11	70.290	-66.115	247	1.06	34.26	5.30	hard	-22.3	no	0.55	2.61	0.23	
150_11	72.739	-79.921	130	-1.36	33.19	7.03	hard	-24.4	no	0.16	1.80	0.33	
160_11	72.670	-78.577	726	1.07	34.39	4.79	soft	-22.8	no	1.36	12.02	0.82	
310F_11	71.299	-97.604	165	-1.18	32.92	5.58	hard	-22.5	yes	0.45	2.49	0.26	
312_11	69.173	-100.755	70	-1.22	30.30	8.01	soft	-23.1	no	0.40	7.55	1.17	
13_11	70.777	-134.376	70	-1.06	32.08	7.68	soft	-26.1	no	1.94	10.79	1.45	
18_11	70.736	-133.916	71	-1.21	32.19	7.78	soft	-26.0	no	1.73	12.97	3.25	
19_11	70.718	-133.639	68	-1.31	32.33	7.21	soft	-26.3	no	1.85	9.12	1.89	
20_11	70.663	-134.774	53	-1.21	32.16	7.40	soft	-26.1	no	1.71	17.97	2.55	
21_11	70.816	-134.610	72	-1.07	31.95	8.14	soft	-26.7	no	0.27	8.24	1.17	
22_11	70.671	-133.364	56	-0.98	32.08	7.95	soft	-26.6	no	0.74	15.09	1.94	
23_11	70.724	-132.873	50	0.16	31.55	8.62	soft	-27.2	no	1.46	3.47	17.74	
24_11	70.793	-132.686	60	-0.91	32.14	7.67	soft	-26.2	no	2.12	18.19	6.14	
25_11	70.577	-133.250	54	-0.98	31.93	8.50	soft	na	no	na	na	na	
26_11	70.679	-132.648	34	-1.18	32.31	6.71	soft	-26.1	no	1.63	30.69	6.88	
27_11	70.775	-132.187	54	-0.89	32.01	7.99	soft	-26.1	no	2.36	12.06	4.35	
28_11	70.858	-132.376	61	-1.07	32.25	7.18	soft	-26.3	no	2.13	20.90	6.90	
29_11	71.016	-132.704	66	-1.16	32.32	6.89	soft	-27.4	no	1.49	11.07	1.83	
405_11	70.630	-123.068	608	0.36	34.78	6.12	soft	-24.8	yes	1.35	2.06	0.06	
420_11	71.050	-128.520	35	-0.87	31.65	9.43	hard	-27.4	yes	0.44	4.63	0.60	
437_11	71.828	-126.505	239	0.13	34.65	6.01	hard	-24.9	yes	0.64	2.49	0.09	
438_11	70.588	-127.613	94	-1.33	33.32	6.39	soft	-28.3	no	1.00	4.53	0.73	
460_11	71.073	-130.577	48	-1.04	32.20	7.68	soft	-26.1	no	1.63	12.26	2.65	
466_11	71.662	-130.788	495	0.35	34.85	6.82	soft	-26.1	no	1.58	2.39	0.06	
115_11	76.330	-71.146	647	0.38	34.37	6.07	soft	-23.1	yes	2.07	11.85	0.54	
GF1_11	71.404	-70.115	364	0.92	34.24	5.68	hard	-23.4	no	0.72	7.27	0.68	
GF2_11	70.760	-72.268	448	0.41	34.06	5.35	soft	-25.1	no	0.30	5.48	0.48	
301_11	74.094	-83.417	665	1.54	34.52	4.97	soft	-22.8	yes	1.79	33.30	3.13	
304_11	74.253	-91.502	315	-0.15	33.84	5.57	soft	-22.4	no	2.48	51.45	6.22	
307_11	74.021	-103.062	368	0.37	34.79	4.93	soft	-22.9	yes	0.54	1.65	0.10	
310M_11	71.693	-101.706	195	-0.45	34.24	4.92	soft	-23.0	no	0.50	1.97	0.19	
312_3_11	69.169	-100.706	66	-0.21	29.86	8.46	soft	-23.6	no	0.43	15.17	4.69	
314_11	69.000	-106.559	119	-0.81	28.82	8.65	soft	-23.9	no	1.18	11.12	1.27	
323_11	74.147	-80.454	789	1.31	34.51	4.82	soft	-23.0	yes	2.27	19.53	1.79	
407_11	71.074	-126.180	408	0.40	34.83	6.72	soft	-25.0	yes	1.57	2.78	0.09	
408_11	71.324	-127.606	207	0.00	34.57	6.08	soft	-25.4	yes	1.68	3.70	0.11	
472_11	72.212	-130.744	1024	-0.07	34.88	6.97	soft	-25.1	no	1.31	1.08	0.02	

References for published 2008-2009 sediment pigment data:

1. Link H, Archambault P, Tamelander T, Renaud PE, Piepenburg D (2011) Spring-to-summer changes and regional variability of benthic processes in the western Canadian Arctic. Polar Biol 34(12): 2025-2038. doi: 10.1007/s00300-011-1046-6.
2. Darnis G, Robert D, Pomerleau C, Link H, Archambault P, et al. (2012) Current state and trends in Canadian Arctic marine ecosystems: II. Heterotrophic food web, pelagic-benthic coupling, and biodiversity. Climatic Change 115(1): 179-205. doi: 10.1007/s10584-012-0483-8.
3. Tremblay JE, Belanger S, Barber DG, Asplin M, Martin J, et al. (2011) Climate forcing multiplies biological productivity in the coastal Arctic Ocean. Geophys Res Lett 38. doi: 10.1029/2011gl048825.
4. Link H, Piepenburg D, Archambault P (2013) Are hotspots always hotspots? The relationship between diversity, resource and ecosystem functions in the Arctic. PloS ONE 8(9): e74077. doi: 10.1371/journal.pone.0074077.

Table S3. Faunal inventory of all megabenthic taxa identified at the lowest possible taxonomic level from 78 stations sampled from 2007 to 2011 across the Canadian Arctic. A total of 527 unique taxa were identified across 13 phyla with 430 at the species level. Taxa are ordered alphabetically within each phylum. Taxonomic identifications were conducted by the first author (Virginie Roy) and by specialists at the Benthic Ecology Lab (Bernard Boucher, Laure de Montety and Lisa Tréau de Coeli; Institut des sciences de la mer de Rimouski, Université du Québec à Rimouski, Rimouski, Québec, Canada). After identification, specimens were transferred to 70 % isopropanol for long-term storage.

Arthropoda	Annelida	Mollusca	Bryozoa	Echinodermata	Cnidaria	Phyla with few taxa
**n = 161**	**n = 122**	**n = 114**	**n = 50**	**n = 43**	**n = 27**	
Acanthonotozoma inflatum	*Abyssoninoe abyssorum*	*Acanthocardia echinata*	*Alcyonidium disciforme*	*Amphiura sundevalli*	*Actinauge verrillii*	**Sipuncula (n = 3)**
Acanthonotozoma serratum	*Abyssoninoe scopa*	*Acmaea* sp.	*Alcyonidium gelatinosum anderssoni*	*Asterias* sp.	*Bolocera* sp.	*Golfingia* sp.
Acanthostepheia malmgreni	*Aglaophamus malmgreni*	*Admete viridula*	*Alcyonidium mamillatum*	*Bathybiaster vexillifer*	Bonneviellidae	*Phascolion* sp.
Aceroides latipes	*Amage auricula*	*Ariadnaria borealis*	*Alcyonidium* sp.*C*	Bourgueticrinina	*Bougainvillia* sp.	Sipunculidae
Aeginina longicornis	*Ampharete acutifrons*	*Astarte borealis*	*Alcyonidium* sp.*E*	*Crossaster papposus*	Ceriantharia	
Amathillopsis spinigera	*Ampharete finmarchica*	*Astarte crenata*	*Arctonula arctica*	*Ctenodiscus crispatus*	*Drifa glomerata*	**Cephalorhyncha (n = 2)**
Amblyops sp.	*Ampharete goesi*	*Astarte montagui*	*Bowerbankia* sp.	*Cucumaria frondosa*	*Edwardsia* sp.	*Priapulopsis bicaudatus*
Ampelisca eschrichtii	*Amphicteis gunneri*	*Aulacofusus brevicauda*	*Bugula* sp.	*Elpidia belyaevi*	Epizoanthidae	*Priapulus caudatus*
Ampelisca macrocephala	*Amphicteis ninonae*	*Axinopsida* sp.	*Callopora craticula*	*Eupyrgus scaber*	*Eudendrium* sp.	
Anonyx compactus	*Amphitrite cirrata*	*Bathyarca glacialis*	*Carbasea carbasea*	*Gorgonocephalus arcticus*	*Filellum serpens*	**Brachiopoda (n = 1)**
Anonyx debruyni	*Aphelochaeta* sp.	*Bathyarca* sp. (< 1.5 cm)	*Cauloramphus intermedius*	*Gorgonocephalus eucnemis*	*Halecium beanii*	Brachiopoda
Anonyx laticoxae	*Aphrodita aculeata*	*Bathypolypus arcticus*	*Cellepora smitti*	*Hathrometra tenella*	*Hormathia digitata*	
Anonyx lilljeborgii	*Apomatus globifer*	*Beringius ossianius*	*Cheilopora sincera*	*Heliometra glacialis*	*Lafoea dumosa*	**Entoprocta (n = 1)**
Anonyx nugax	*Apomatus similis*	*Boreotrophon clathratus*	*Cribrilina spitzbergensis*	*Henricia* sp.	*Lafoeina maxima*	Entoprocta
Anonyx pacificus	*Asychis biceps*	*Boreotrophon pacificus*	*Crisia* sp.	*Hymenaster pellucidus*	Nephtheidae	
Apherusa sp.	*Axionice flexuosa*	*Boreotrophon truncatus*	*Cystisella saccata*	*Icasterias panopla*	*Obelia longissima*	**Nemertea (n = 1)**
Arctolembos arcticus	*Axionice maculata*	*Buccinum angulosum*	*Dendrobeania levinseni*	Korethrasteridae	*Obelia loveni*	Nemertea
Arcturus baffini	*Brada inhabilis*	*Buccinum belcheri*	*Doryporella spathulifera*	*Leptasterias groenlandica*	*Paraedwardsia arenaria*	
Arcturus baffini tuberosus	*Brada villosa*	*Buccinum ciliatum*	*Escharella dijmphnae*	*Lethasterias* sp.	*Schuchertinia allmanii*	**Platyhelminthes (n = 1)**
Argis dentata	*Branchiomma* sp.	*Buccinum ciliatum sericatum*	*Escharoides jacksoni*	*Lophaster furcifer*	Scleractinia	Platyhelminthes
Aristias tumidus	*Bylgides elegans*	*Buccinum cyaneum*	*Escharopsis rosacea*	Molpadiidae	*Sertularia* sp.	
Arrhis phyllonyx	*Bylgides groenlandicus*	*Buccinum hydrophanum*	*Escharopsis sarsi*	*Myriotrochus rinkii*	*Stegopoma plicatile*	**Porifera (n = 1)**
Atylus carinatus	*Bylgides sarsi*	*Buccinum micropoma*	*Eucratea loricata*	*Ophiacantha bidentata*	*Stephanauge* sp.	Porifera
Atylus smittii	*Calliobdella* sp.	*Buccinum plectrum*	*Eucratea loricata arctica*	*Ophiocten sericeum*	Subsessiliflorae	
Balanus balanus	Chaetopteridae	*Buccinum polare*	*Flustra* sp.	*Ophiopholis aculeata*	*Symplectoscyphus tricuspidatus*	
Balanus crenatus	*Chaetozone* sp.	*Buccinum scalariforme*	*Hippoporina reticulatopunctata*	*Ophiopleura borealis*	*Thuiaria* sp.	
Balanus rostratus	*Chone duneri*	Chaetodermatida	Idmoneidae	*Ophiopus arcticus*	Zoanthidae	
Birsteiniamysis inermis	*Chone gracilis*	*Chlamys islandica*	*Kinetoskias arborescens*	*Ophioscolex glacialis*		
Boreomysis arctica	*Chone infundibuliformis*	*Ciliatocardium ciliatum ciliatum*	*Lichenopora crassiuscula*	*Ophiura robusta*		
Boreonymphon abyssorum	*Cirratulus cirratus*	*Clione limacina*	Membraniporidae	*Ophiura sarsii*		
Boreonymphon ossiansarsi	*Cistenides hyperborea*	*Colus holboelli*	*Myriapora* sp.	*Poliometra prolixa*		
Boreonymphon robustum	*Diplocirrus* sp.	*Colus islandicus*	*Myriozoella plana*	*Pontaster tenuispinus*		
Byblis erythrops	*Dipolydora socialis*	*Colus latericeus*	*Patinella* sp.	*Porania (Porania) pulvillus*		
Byblis gaimardii	*Ephesiella* sp.	*Colus pubescens*	*Porella sacata*	*Poraniomorpha tumida*		
Bythocaris gracilis/payeri	*Eteone flava/longa*	*Colus sabini*	*Porella smitti*	*Pourtalesia* sp.		
Caecognathia elongata	*Euchone analis*	*Cryptonatica affinis*	*Posterula sarsii*	*Psilaster andromeda*		
Caecognathia stygia	*Euchone elegans*	*Curtitoma decussata*	*Pseudoflustra anderssoni*	*Psolus fabricii*		
Calathura brachiata	*Euchone papillosa*	*Curtitoma incisula*	*Pseudoflustra sinuosa*	*Pteraster militaris*		
Caprella linearis	*Eucranta villosa*	*Cuspidaria glacialis*	*Pseudoflustra solida*	*Pteraster obscurus*		
Centromedon calcaratus	*Eunereis longissima*	*Cuspidaria rostrata*	*Rhamphostomella costata*	*Pteraster pulvillus*		
Colossendeis angusta	*Eunoe nodosa*	*Cyclopecten hoskynsi*	*Rhamphostomella ovata*	*Stegophiura nodosa*		
Colossendeis proboscidea	*Eunoe oerstedi*	*Cylichna alba*	*Sarsiflustra abyssicola*	*Strongylocentrotus* sp.		
Diastylis echinata	*Euphrosine borealis*	*Cylichna occulta*	*Schizoporella costata*	*Urasterias lincki*		

Diastylis glabra	*Eupolymnia* sp.	*Cyrtodaria siliqua*	*Scrupocellaria* sp.			
Diastylis goodsiri	Fabriciidae	*Diaphana globosa*	*Semibugula birulai*			
Diastylis oxyrhyncha	*Flabelligera affinis*	*Ennucula tenuis*	*Smittina jeffreysi*			
Diastylis rathkei	*Galathowenia oculata*	*Euspira pallida*	*Tegella armifera*			
Diastylis scorpioides	*Gattyana cirrhosa*	*Hiatella arctica*	*Tegella inermis*			
Diastylis spinulosa	*Glycera capitata*	*Lepeta caeca*	*Tricellaria ternata*			
Diastyloides sp.	*Glyphanostomum pallescens*	*Limatula subauriculata*	*Tubulipora* sp.			
Epimeria loricata	*Grubianella klugei*	*Limneria undata*				
Ericthonius punctatus	*Harmothoe extenuata*	*Liocyma fluctuosa*				
Erythrops abyssorum	*Harmothoe rarispina*	*Lyonsia arenosa*				
Erythrops glacialis	*Heteromastus* sp.	*Lyonsiella* sp.				
Eualus fabricii	Hirudinea	*Macoma brota*				
Eualus gaimardii belcheri	*Jasmineira* sp.	*Macoma calcarea*				
Eualus gaimardii gaimardii	*Laonice cirrata*	*Macoma moesta*				
Eudorella emarginata	*Laonome* sp.	*Macoma torelli*				
Eurycope sp.	*Leaena abranchiata*	*Margarites costalis*				
Eurycyde hispida	*Levinsenia gracilis*	*Margarites groenlandicus*				
Eusirus cuspidatus	*Lumbrineris latreilli*	*Margarites groenlandicus umbilicalis*				
Eusirus holmi	*Lumbrineris scopa*	*Margarites olivaceus*				
Eusirus leptocarpus	*Lysippe labiata*	*Margarites sordidus*				
Gnathia sp.	*Macellicephala violacea*	*Megayoldia thraciaeformis*				
Halice abyssi	*Maldane arctica*	*Mitrella* sp.				
Halirages fulvocinctus	*Maldane sarsi*	*Montacuta* sp.				
Halirages qvadridentatus	*Melinna cristata*	*Musculus discors*				
Haliragoides inermis	*Melinna palmata*	*Musculus niger*				
Haploops laevis	*Melinnopsis annenkovae*	*Mya* sp.				
Haploops tubicola	*Melinnopsis arctica*	*Neptunea despecta*				
Hemiarthrus abdominalis	*Monticellina* sp.	*Neptunea heros*				
Hippomedon propinquus	*Myriochele heeri*	*Neptunea intersculpta*				
Hyas alutaceus	*Myriochele olgae*	*Neptunea ithia*				
Hymenodora glacialis	*Myrioglobula malmgreni*	*Nodulotrophon coronatus*				
Hyperia galba	*Nephtys ciliata*	*Nucula pusilla*				
Hyperia spinigera	*Nephtys incisa*	*Nuculana minuta*				
Ichnopus spinicornis	*Nephtys longosetosa*	*Nuculana pernula*				
Ischyrocerus latipes	*Nephtys paradoxa*	Nudibranchia sp.A				
Ischyrocerus megacheir	*Nereis zonata*	Nudibranchia sp.B				
Jassa sp.	*Nicomache lumbricalis*	Nudibranchia sp.C				
Lebbeus polaris	*Nothria conchylega*	*Odostomia* sp.				
Lepidepecreella sp.	*Notomastus* sp.	*Oenopota bicarinata*				
Lepidepecreum serratum	Oligochaeta	*Oenopota cinerea*				
Lepidepecreum umbo	*Ophelina aulogaster*	*Oenopota obliqua*				
Leucon (Leucon) nasica	*Ophelina cylindricaudata*	*Pandora glacialis*				
Leucon (Leucon) nasicoides	*Oriopsis* sp.	*Panomya norvegica*				
Leucon (Leucon) nathorsti	*Owenia borealis*	*Periploma* sp.				
Lophogastridae	*Owenia fusiformis*	*Philine finmarchica*				
Melita dentata	*Owenia polaris*	*Philine quadrata*				
Melita formosa	*Paradiopatra quadricuspis*	*Piliscus commodus*				
Metacaprella horrida	*Paramphitrite tetrabranchia*	*Plicifusus kroeyeri*				
Metopa spitzbergensis	*Paranaitis wahlbergi*	*Portlandia arctica*				
Michthyops arctica	*Paraonis gracilis*	*Portlandia intermedia*				
Munnopsis typica	*Petaloproctus tenuis*	*Propebela turricula/nobilis*				
Munnopsurus giganteus	*Pholoe* sp.	*Rossia palpebrosa*				
Nebalia sp.	*Phyllodoce (Anaitides) groenlandica*	*Scabrotrophon fabricii*				
Neohela monstrosa	*Phyllodoce mucosa*	*Semirossia tenera*				
Neopleustes pulchellus	*Pista elongata*	*Serripes groenlandicus*				
Nototropis smitti	*Polycirrus* sp.	*Similipecten greenlandicus*				

Nymphon brevirostre	*Polyphysia baffinensis*	*Siphonodentalium lobatum*				
Nymphon elegans	*Polyphysia crassa*	*Solariella* sp.				
Nymphon grossipes	*Potamilla neglecta*	*Tachyrhynchus erosus*				
Nymphon hirtipes	*Prionospio cirrifera*	*Testudinalia testudinalis*				
Nymphon leptocheles	*Prionospio steenstrupi*	Thraciidae				
Nymphon longitarse	*Protis arctica*	*Thyasira gouldi*				
Nymphon macronyx	*Protula tubularia*	*Trichotropis bicarinata*				
Nymphon macrum	*Pterolysippe vanelli*	Turridae				
Nymphon serratum	*Sabellides borealis*	*Turrisipho lachesis*				
Nymphon sluiteri	*Sabellides octocirrata*	*Velutina* sp.				
Nymphon stroemi	*Samythella neglecta*	*Volutomitra groenlandica*				
Oediceros sp.	*Scalibregma inflatum*	*Volutopsius norwegicus*				
Onisimus barentsi	*Scoletoma fragilis*	*Yoldia hyperborea*				
Onisimus brevicaudatus	*Scoletoma impatiens*	*Yoldiella frigida*				
Onisimus edwardsi	*Scoletoma tetraura*	*Yoldiella lenticula*				
Onisimus litoralis	*Scoloplos armiger*					
Orchomenella sp.	*Sphaerodorum* sp.					
Orchomenopsis obtusus	*Spio* sp.					
Pandalus montagui	*Spirorbis* sp.					
Paralibrotus setosus	*Terebellides gracilis*					
Paramphithoe hystrix	*Terebellides stroemii*					
Paramphithoe polyacantha	*Tharyx* sp.					
Paratryphosites abyssi	*Thelepus cincinnatus*					
Pardalisca abyssi						
Pardalisca cuspidata						
Parerythrops spectabilis						
Paroediceros intermedius						
Paroediceros lynceus						
Phippsiella similis						
Pleustes (Pleustes) panoplus						
Pontoporeia femorata						
Praunus flexuosus						
Protomedeia fasciata						
Protomedeia grandimana						
Pseudomma affine						
Pseudomma roseum						
Quasimelita quadrispinosa						
Rhachotropis aculeata						
Rhachotropis macropus						
Rhachotropis oculata						
Rostroculodes borealis						
Rozinante fragilis						
Sabinea septemcarinata						
Saduria entomon						
Saduria sabini						
Saduria sibirica						
Scalpellum sp.						
Sclerocrangon ferox						
Socarnes bidenticulatus						
Spirontocaris intermedia						
Spirontocaris phippsii						
Spirontocaris spinus						
Stegocephalus inflatus						
Synidotea bicuspida						
Synidotea marmorata						
Themisto abyssorum						

Themisto libellula						
Tmetonyx acutus						
Tmetonyx cicada						
Tmetonyx similis						
Tritella pilimana						
Unciola leucopis						

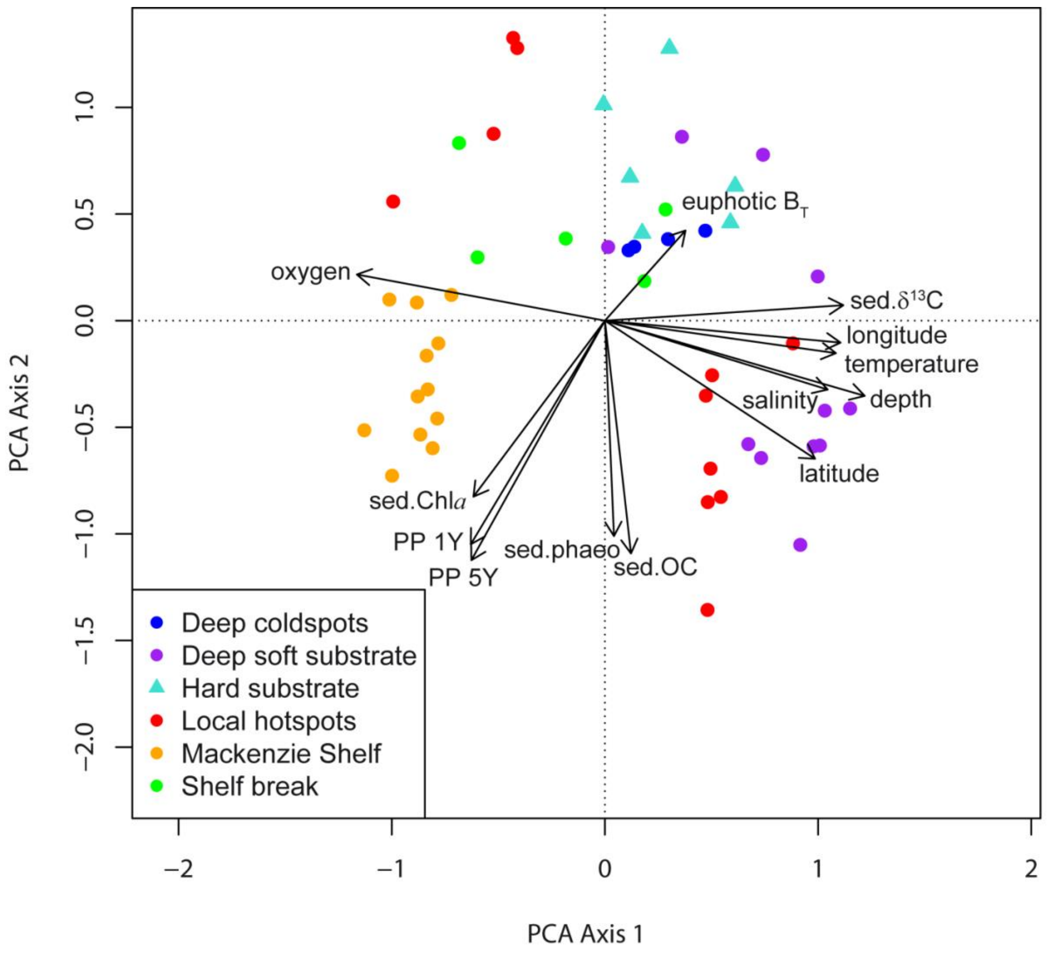

Figure S1. Principal Component Analysis (PCA) plot showing the multivariate variation among 50 stations in terms of environmental variables. Vectors indicate the direction and strength of each environmental variable to the overall distribution. Colored symbols correspond to the six megabenthic community clusters defined in this study. The first two principal axes explained 64 % of the variance (λ_1=5.1 and λ_2=3.2).

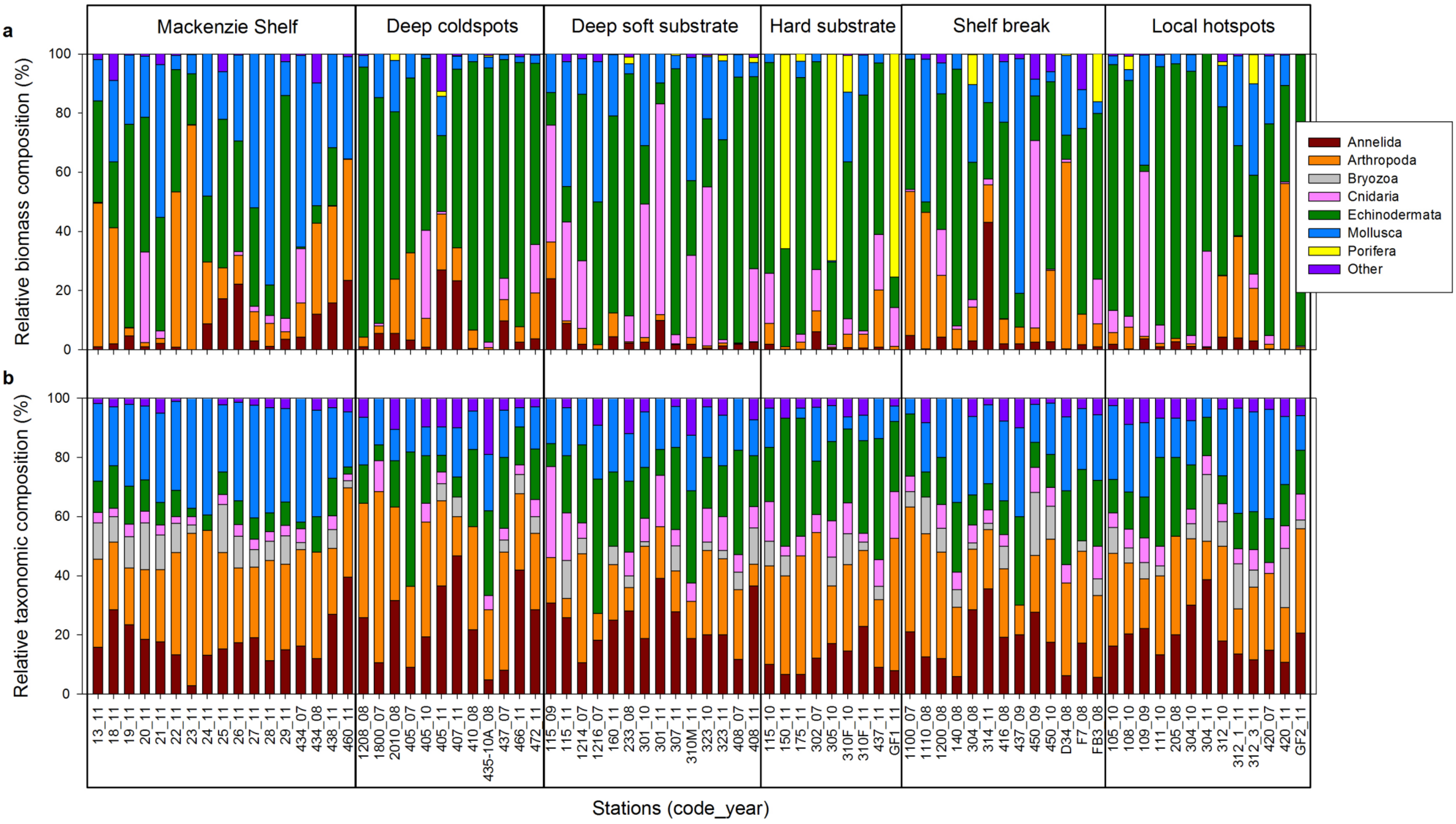

Figure S2. Station-based variation in mean relative (%) (a) biomass composition, and (b) taxonomic richness composition for the main phyla sampled across all community clusters. Stations are grouped by community clusters and are organised within clusters by numeric order. Station codes correspond to ArcticNet expedition labels.
